# Self-Assembly of Chiral Porous Metal–Organic Polyhedra from Trianglsalen Macrocycles

**DOI:** 10.1021/jacs.4c04928

**Published:** 2024-06-11

**Authors:** Donglin He, Heng Ji, Tao Liu, Miao Yang, Rob Clowes, Marc A. Little, Ming Liu, Andrew I. Cooper

**Affiliations:** † Materials Innovation Factory and Department of Chemistry, University of Liverpool, 51 Oxford Street, Liverpool L7 3NY, U.K.; ‡ ZJU-Hangzhou Global Scientific and Technological Innovation Center, Hangzhou 311215, China; § Department of Chemistry, Zhejiang University, Hangzhou 310027, China; ∥ Leverhulme Research Centre for Functional Materials Design, University of Liverpool, 51 Oxford Street, Liverpool L7 3NY, U.K.; ⊥ Institute of Chemical Sciences, School of Engineering and Physical Sciences, Heriot-Watt University, Edinburgh EH14 4AS, U.K.

## Abstract

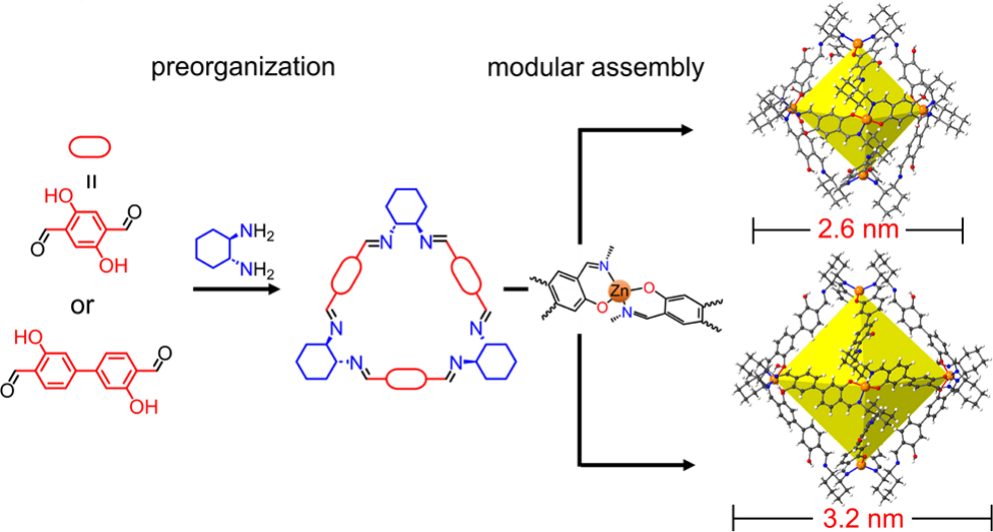

Metal–organic polyhedra (MOPs) can exhibit tunable porosity and functionality, suggesting potential for applications such as molecular separations. MOPs are typically constructed by the bottom-up multicomponent self-assembly of organic ligands and metal ions, and the final functionality can be hard to program. Here, we used trianglsalen macrocycles as preorganized building blocks to assemble octahedral-shaped MOPs. The resultant MOPs inherit most of the preorganized properties of the macrocyclic ligands, including their well-defined cavities and chirality. As a result, the porosity in the MOPs could be tuned by modifying the structure of the macrocycle building blocks. Using this strategy, we could systematically enlarge the size of the MOPs from 26.3 to 32.1 Å by increasing the macrocycle size. The family of MOPs shows experimental surface areas of up to 820 m^2^/g, and they are stable in water. One of these MOPs can efficiently separate the rare gases Xe from Kr because the prefabricated macrocyclic windows of MOPs can be modified to sit at the Xe/Kr size cutoff range.

## Introduction

MOPs are discrete coordination complexes with polyhedral structures that are synthesized through the self-assembly of metal ions and organic ligands.^1^ MOPs often have good solubility in organic solvents and aqueous solutions and have tunable cavity sizes and permanent porosities. MOPs have, therefore, attracted attention for applications in host–guest chemistry,^2,3^ gas separation,^4−8^ and gas storage.^9^

**Figure 1:**
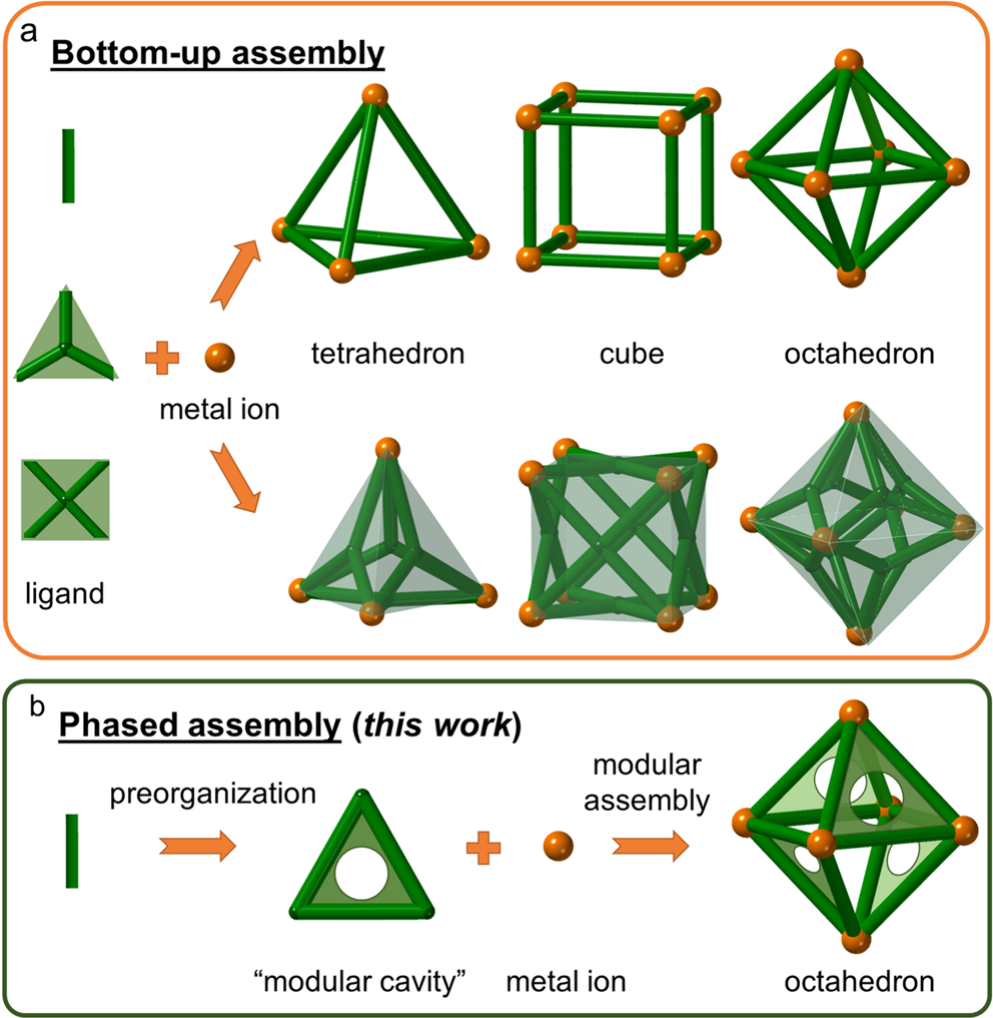
Synthesis of MOPs by (a) bottom-up assembly of organic ligands and metal ions and (b) phased assembly via preorganized macrocyclic building blocks (this work).

Conventionally, MOPs are constructed by bottom-up assembly approaches (Figure 1a). Such approaches enable MOPs with different shapes to be isolated by tuning the bonding geometry and directionality between organic ligands and metal nodes.^10,11^ For example, as shown in Figure 1a, linear bidentate ligands^11^ can be assembled using metal ions with different coordination geometries into Platonic solids with tetrahedron, cube, and octahedron shapes. However, subtle changes to the ligand geometry can profoundly affect the assembly of MOPs,^12^ and it is difficult to obtain isostructural families of MOPs in the same way that can be achieved by varying the organic ligands in the case of isoreticular frameworks, such as metal–organic frameworks (MOFs).^13^ Here, we propose a phased assembly strategy that uses preorganized, intrinsically porous macrocycles as ligands for the modular construction of MOPs, thus allowing finer control over the porous properties of the assembled products. First, a rigid trigonal macrocycle with an intrinsic cavity is prepared (Figure. 1b), which is then used as a ligand to form MOPs. This approach ensures that the main structural features of the macrocycles are retained in the self-assembled MOPs. Macrocycles with intrinsic cavities, such as calixarenes,^14^ calix[4]resorcinarenes,^15^ porphyrins,^16^ and calixsalens,^17^ have been used as ligands to construct MOPs in recent years. Here, we focused on rigid trianglimine macrocycles as preorganized building blocks to form MOPs.

Rigid organic macrocycles have been investigated for solid-state molecular separations and can show good selectivity as a result of their rich host–guest chemistry. For example, Barbour et al. showed that a trianglimine macrocycle absorbs atmospheric water molecules reversibly.^18^ Also, we reported a trianglimine macrocycle with high selectivity for ethyl acetate over ethanol based on ideal adsorption solution theory.^19^

## Results and Discussion

**Figure 2:**
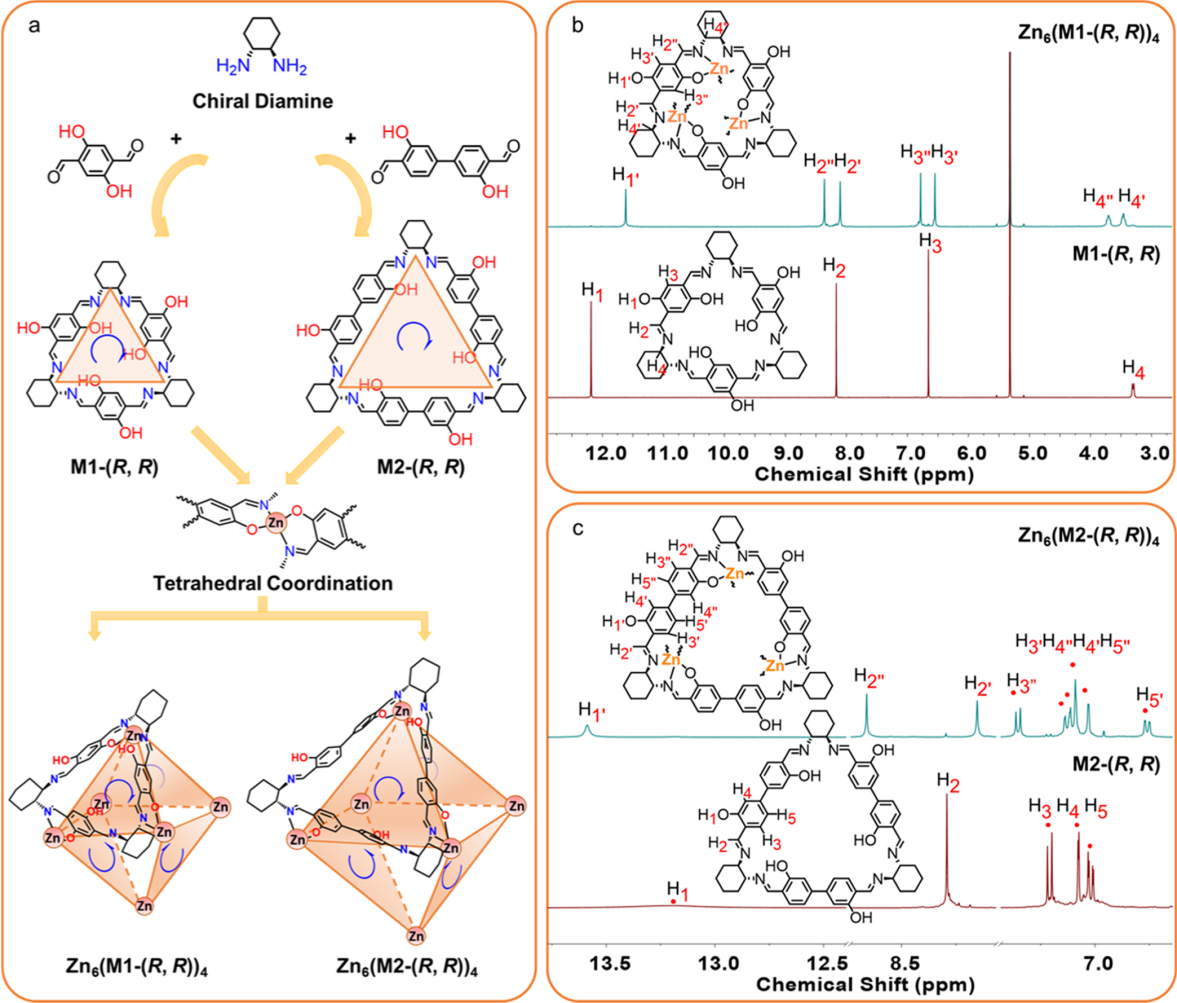
(a) Scheme showing the preorganization of **M1**-(*R, R*) and **M2**-(*R, R*) for the self-assembly of Zn_6_(**M1**-(*R, R*))_4_ and Zn_6_(**M2**-(*R, R*))_4_. The orange triangles highlight the intrinsic cavities of **M1**-(*R, R*) and **M2**-(*R, R*) in the structures. ^1^H NMR spectra (400 MHz, CD_2_Cl_2_, 293 K) of (b) **M1**-(*R, R*) and Zn_6_(**M1**-(*R, R*))_4_, and (c) **M2**-(*R, R*) and Zn_6_(**M2**-(*R, R*))_4_.

Synthesis and Characterization of MOPs from Trianglsalen Macrocycles: Two chiral trianglsalen macrocycles, **M1**-(*R*, *R*) and **M2**-(*R*, *R*), with different-sized intrinsic cavities, were synthesized for this study (Figure 2a). We synthesized **M1**-(*R*, *R*) using a previously reported procedure by condensing 2,5-dihydroxyterephthalaldehyde with (1*R*, 2*R*)-diaminocyclohexane in chloroform (CHCl_3_) at room temperature (RT) to afford the purified product in 80% yield.^20^ We synthesized **M2**-(*R*, *R*) by condensing 3,3′-dihydroxy[1,1′-biphenyl]-4,4′-dicarboxaldehyde with (1*R*, 2*R*)-diaminocyclohexane in a 1:1 tetrahydrofuran (THF):ethanol (EtOH) solvent mixture at RT to afford the purified product in a yield of 67%. It should be noted that **M2**-(*R*, *R*) is a [3 + 3] product confirmed by mass spectroscopy (MS) with an *m*/*z* of 961.465 (Figure S8) and is the kinetic product of the reaction. Dissolving **M2**-(*R*, *R*) in THF results in this [3 + 3] product transforming slowly into a larger [4 + 4] macrocycle, **M3**-(*R*, *R*), which we crystallized by slowly diffusing EtOH into the THF solution. We confirmed the structure of **M3**-(*R*, *R*) by NMR spectroscopy, MS, and element analysis (EA) (see SI Section 2.3 for full details). However, due to the poorer solubility of **M3**-(*R*, *R*) in organic solvents than **M1**-(*R*, *R*) and **M2**-(*R*, *R*) and its different structural geometry, we did not investigate the self-assembly of **M3**-(*R*, *R*) into MOPs in this study.

To synthesize MOPs using **M1**-(*R, R*) and **M2**-(*R, R*), we used Zn(CH_3_COO)_2_·2H_2_O as the metal source, noting that the acetate anions can facilitate deprotonation of the hydroxy group of **M1**-(*R*, *R*) and **M2**-(*R*, *R*). We initially used ^1^H NMR spectroscopy to investigate the molecular composition and structure of the products from the reactions in solution. The products from both reactions are slightly soluble in CHCl_3_ and CH_2_Cl_2_, and their ^1^H NMR spectra were recorded in CD_2_Cl_2_ and compared with unreacted **M1**-(*R, R*) and **M2**-(*R, R*). As shown in Figures 2b and S14 for the H_1_ singlet integral, the hydroxyl proton singlet (H_1_′) in Zn_6_(**M1**-(*R, R*))_4_ indicates half of the salen units are not deprotonated and do not coordinate with Zn(II) cations. The imine proton singlet (H_2_) of **M1**-(*R, R*) splits into two singlets (H_2_′, H_2_″) in Zn_6_(**M1**-(*R, R*))_4_, with the H_2_″ singlet shifted downfield due to coordination of the nitrogen donors to the Zn(II) cations (Figure 2b). Likewise, the aromatic proton singlet (H_3_) of **M1**-(*R, R*) splits into two aromatic singlets (H_3_′, H_3_″) in Zn_6_(**M1**-(*R, R*))_4_, with the aromatic H_3_″ singlet shifted downfield due to its close proximity to the deprotonated phenolic hydroxyl group (Figure 2b). In contrast, the H_2_′ imine singlet and H_3_′ aromatic singlet shift upfield slightly (Figure 2b). As shown in Figures 2c and S19, similar signal shifts and splitting of singlets were found comparing the NMR spectra of **M2**-(*R, R*) with Zn_6_(**M2**-(*R, R*))_4_, with the imine signal (H_2_) and aromatic signals (H_3_, H_4_, H_5_) of **M2**-(*R, R*) observed to split into the downfield shifted singlets H_2_″, H_3_″, H_4_″, and H_5_″ and the upfield shifted singlets H_2_′, H_3_′, H_4_′, and H_5_′. In conclusion, the NMR data indicates the formation of symmetrical MOPs in solution that fit with the octahedral structure of Zn_6_(**M1**-(*R, R*))_4_ and Zn_6_(**M2**-(*R, R*))_4_ shown in Figure 2, which was later confirmed by single-crystal X-ray diffraction (SC-XRD).

We also used matrix-assisted laser desorption ionization-time-of-flight mass spectrometry (MALDI-TOF MS) to investigate the composition of the reaction products. In the MALDI-TOF MS, from the reaction with **M1**-(*R, R*), we observed a peak with an *m*/*z* of 3311.712 that we assigned to the product [Zn_6_(**M1**-(*R, R*))_4_H^+^]^1+^, which has a calculated mass of 3312.146. The MALDI-TOF MS data, therefore, confirms the formation of the Zn_6_(**M1**-(*R, R*))_4_ complex (Figure S16). In the MALDI-TOF MS spectrum from the reaction with **M2**-(*R, R*), we assigned the peaks *m*/*z* of 2179.570 and 2242.915 to the products [Zn_4_(**M2**-(*R, R*))_2_H^+^]^1+^ and [Zn_5_(**M2**-(*R, R*))_2_H^+^]^1+^, respectively. However, we did not observe an *m*/*z* peak at 4224.888, which we calculated for the isostructural [Zn_6_(**M2**-(*R, R*))_4_H^+^]^1+^ product (Figure S20).

### Crystal Structures of Zn_6_(M1-(*R, R*))_4_ and Zn_6_(M2-(*R, R*))_4_

**Figure 3:**
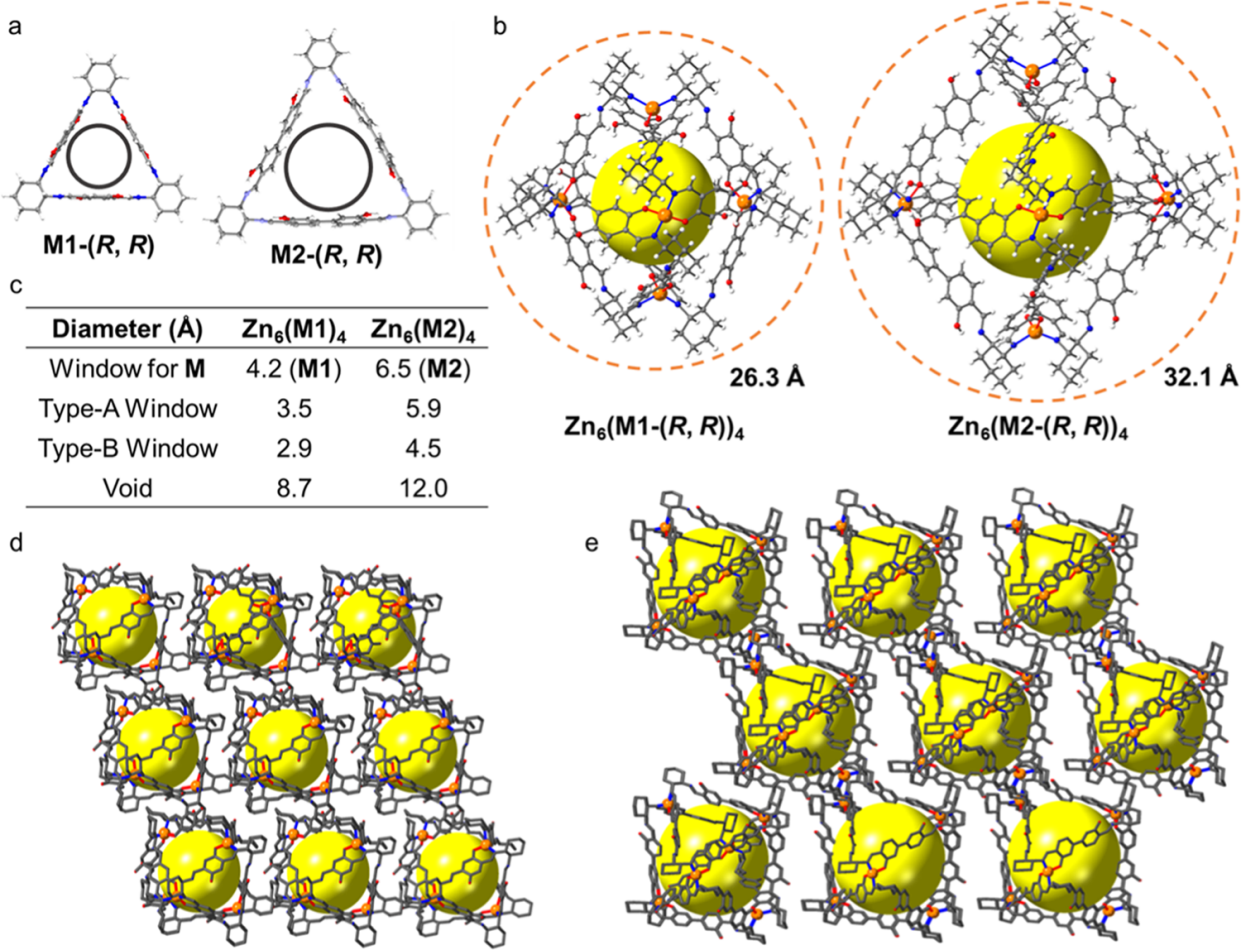
(a) Structures of **M1**-(*R, R*) and **M2**-(*R, R*) simulated and optimized by the wB97XD^22^/def2-svp^23^ level implemented in Gaussian16.^24^ (b) Crystal structures of Zn_6_(**M1**-(*R, R*))_4_ and Zn_6_(**M2**-(*R, R*))_4_; showing the arrangement of the four type-A windows from the preorganized trianglsalen macrocycles and the type-B windows that occupy the four other octahedron faces. (c) Structural parameters calculated with *py-window* for **M1**-(*R, R*), **M2**-(*R, R*), Zn_6_(**M1**-(*R, R*))_4_, and Zn_6_(**M2**-(*R, R*))_4_. Crystal packing images of (d) Zn_6_(**M1**-(*R, R*))_4_ and (e) Zn_6_(**M2**-(*R, R*))_4_; the large yellow spheres represent the intrinsic cavities of the MOPs, H atoms are omitted for clarity. Orange, Zn; blue, N; red, O; gray, C; white, H.

We recrystallized the as-synthesized **M1**-(*R, R*) product from CH_3_CN for SC-XRD analysis.^21^ Unfortunately, we did not obtain a single crystal of **M2**-(*R, R*) because it transformed into the thermodynamic [4 + 4] product, **M3**-(*R, R*), during the crystallization. We, therefore, generated a model for **M2**-(*R, R*) that was simulated and optimized by the wB97XD^22^/def2-svp^23^ level implemented in Gaussian16.^24^ The molecular structures of **M1**-(*R, R*) and **M2**-(*R, R*) are shown in Figure 3a, along with the intrinsic cavity sizes of these molecules.

We obtained red single crystals of Zn_6_(**M1**-(*R, R*))_4_ (Figure S22a) that were suitable for SC-XRD by slowly diffusing acetonitrile (CH_3_CN) vapor into an *N*,*N*-diethylformamide (DEF) solution containing the reagents. In a related procedure, we obtained yellow single crystals of Zn_6_(**M2**-(*R, R*))_4_ from a DEF solution after leaving the reagents undisturbed at RT for 3 days (Figure S22c). The octahedral geometries of the resulting MOPs shown in Figure 2a were further confirmed by SC-XRD (Figure 3). The SC-XRD analysis revealed that the solvated single crystals of Zn_6_(**M1**-(*R, R*))_4_ and Zn_6_(**M2**-(*R, R*))_4_ have a trigonal *R*3 space group symmetry (see Table S1 for full refinement details). Further analysis of the SC-XRD structures revealed that Zn_6_(**M1**-(*R, R*))_4_ and Zn_6_(**M2**-(*R, R*))_4_ are isostructural and have octahedron geometries (Figure 3). Interestingly, Zn_6_(**M1**-(*R, R*))_4_ and Zn_6_(**M2**-(*R, R*))_4_ contain four triply deprotonated trianglsalen macrocycles coordinated to six octahedrally arranged Zn(II) cations. Each MOP contains eight windows through which guests can access the intrinsic cavities on each face of the octahedron (Figure 3b). Four of these windows are prescribed by the four chiral, trianglsalen macrocycles, which we refer to as type-A windows. The four other windows, which we refer to as type-B windows, occupy four other octahedron faces. To compare the SC-XRD structures of Zn_6_(**M1**-(*R, R*))_4_ and Zn_6_(**M2**-(*R, R*))_4_, we calculated the maximum diameter of a molecule (*D*_max_), the window diameter (*D*_type-A_ or *D*_type-B_), the intrinsic void diameter (*D*_void_), and the spherical pore volume (*V*_void_) using *py-window.*(25) As shown in Figure 3b, the *D*_max_ values are 26.3 Å for Zn_6_(**M1**-(*R, R*))_4_ and 32.1 Å for Zn_6_(**M2**-(*R, R*))_4_. As shown in Figure 3c, the larger intrinsic cavity of **M2**-(*R, R*) than **M1**-(*R, R*) results in the average *D*_type-A_ increasing from 3.5 Å in Zn_6_(**M1**-(*R, R*))_4_ to 5.9 Å in Zn_6_(**M2**-(*R, R*))_4_. This also results in the average *D*_type-B_ increasing from 2.9 Å in Zn_6_(**M1**-(*R, R*))_4_ to 4.5 Å in Zn_6_(**M2**-(*R, R*))_4_. The intrinsic MOP cavities, which we highlight using yellow spheres in Figure 3b, show that the *D*_void_ is 8.7 Å for Zn_6_(**M1**-(*R, R*))_4_ and 12.0 Å for Zn_6_(**M2**-(*R, R*))_4_, which indicates that their *V*_void_ sizes are 350 and 916 Å^3^, respectively.

We anticipated that the 3-D porous supramolecular architectures of the MOPs and their crystal structures were likely stabilized by solvent molecules filling the crystal voids. We have shown the crystal packing images of Zn_6_(**M1**-(*R, R*))_4_ and Zn_6_(**M2**-(*R, R*))_4_ in Figure 3d,e, using yellow spheres to highlight the intrinsic MOP cavities. In addition to these intrinsic MOP cavities, the Zn_6_(**M1**-(*R, R*))_4_ and Zn_6_(**M2**-(*R, R*))_4_ crystal structures contain large solvent-accessible extrinsic voids, and the combined solvent-accessible volumes in Zn_6_(**M1**-(*R, R*))_4_ and Zn_6_(**M2**-(*R, R*))_4_, as calculated by *Platon*,^100^ are 43.0 and 64.7%, respectively. These large solvent-accessible values indicate that both MOP crystal structures would be highly porous if they could remain intact after being activated by removing the crystallization solvents.

### Chirality Inheritance and Amplification

**Figure 4:**
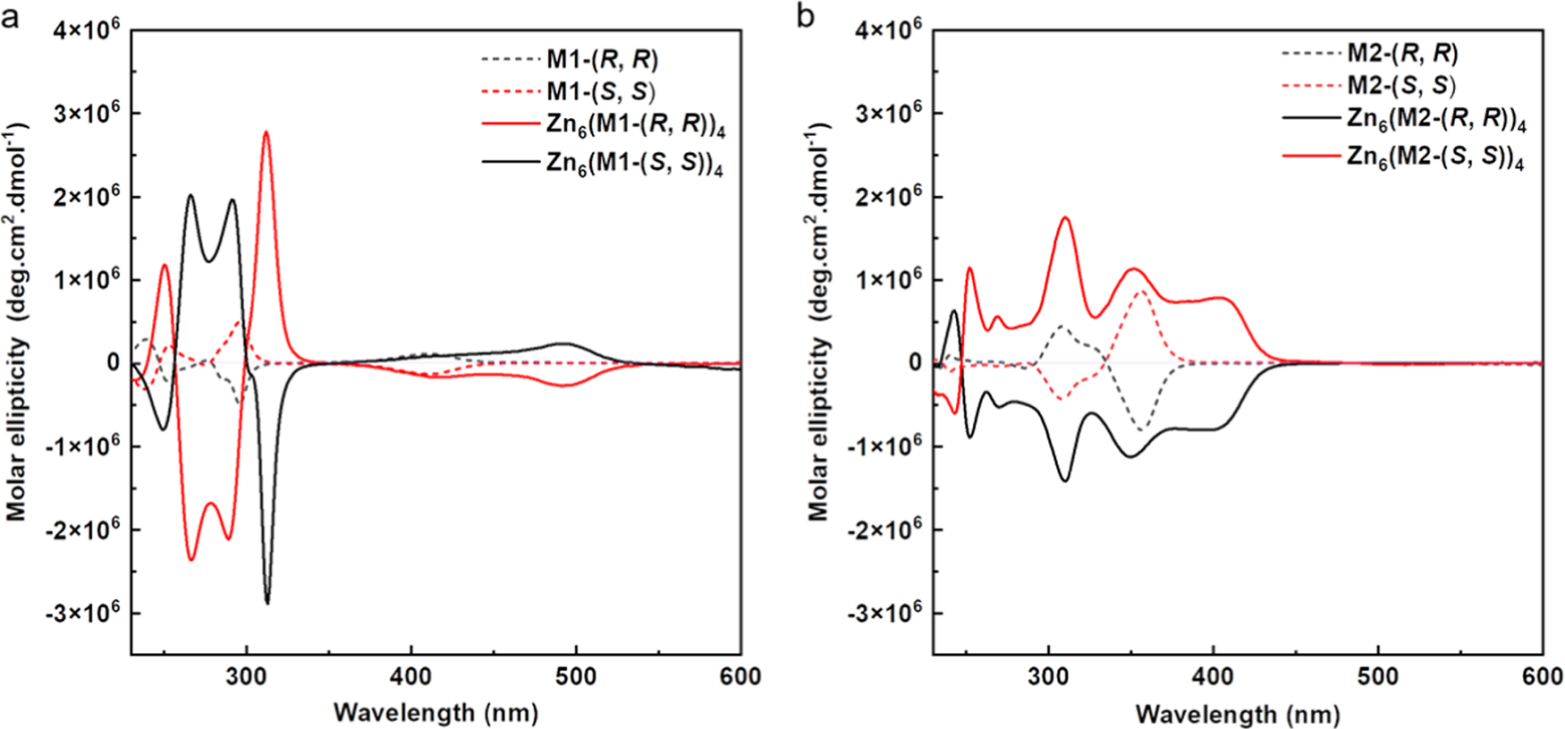
CD spectra in CH_2_Cl_2_ of (a) **M1**-(*R*, *R*) and Zn_6_(**M1**-(*R*, *R*))_4_, and (b) **M2**-(*R*, *R*) and Zn_6_(**M2**-(*R*, *R*))_4_.

We next studied if incorporating homochiral trianglsalen macrocycles into MOPs afforded enantiopure chiral MOPs because of the predetermined, “hard” chirality of the trianglsalen macrocycles.^26^ As shown in Figure 4, the circular dichroism (CD) spectra of the macrocycle building blocks and the resulting MOPs were recorded in CH_2_Cl_2_. The solution CD spectra of unreacted -(*R, R*) and -(*S*, *S*) trianglsalen macrocycles are mirror images of each other, and the assembled -(*R, R*) and -(*S*, *S*) exhibit the same CD behavior, which indicates that the MOPs inherit the chirality from the trianglsalen macrocycles. The strongly enhanced CD signals of the MOPs compared with the CD of unreacted trianglsalen macrocycles suggest chiral amplification occurs after self-assembly of the MOPs in solution. The absorption bands of MOPs in the UV–vis spectra (Figure S23) have red-shifts compared with unreacted trianglsalen macrocycles, which we attribute to the metal-to-ligand charge transfer (MLCT) transition between Zn(II) cations and trianglsalen macrocycle ligands.

### Stability of Zn_6_(M1-(*R*, *R*))_4_ and Zn_6_(M2-(*R*, *R*))_4_ in the Solid State

Before activating the DEF-solvated crystals of Zn_6_(**M1**-(*R, R*))_4_, we first exchanged the DEF crystallization solvent with the more volatile solvent, CH_3_CN, to assist with desolvating the crystals at lower temperatures. As shown in Figure S24, the simulated PXRD pattern of the Zn_6_(**M1**-(*R, R*))_4_ single-crystal structure and the experimental PXRD pattern after exchanging the crystallization solvents with CH_3_CN are comparable and indicate that the structure of Zn_6_(**M1**-(*R, R*))_4_ remains crystalline after exchanging the DEF solvent. We then attempted to activate the CH_3_CN-exchanged crystals of Zn_6_(**M1**-(*R, R*))_4_ thermally and recorded PXRD patterns while heating the sample from 298 to 438 K. The variable-temperature PXRD patterns revealed that heating CH_3_CN-exchanged Zn_6_(**M1**-(*R, R*))_4_ broadened the PXRD peaks slightly but that the sample remained partially crystalline (Figure S24). We also investigated activating the CH_3_CN-exchanged crystals of Zn_6_(**M1**-(*R, R*))_4_ at 373 K under vacuum for 12 h, which resulted in the peaks in the PXRD pattern changing and becoming broad (Figure S25). We explored two conditions to desolvate the DEF-solvated crystals of Zn_6_(**M2**-(*R*, *R*))_4_. First, we exchanged the DEF crystallization solvent with CH_3_CN and attempted to activate the CH_3_CN-exchanged crystals of Zn_6_(**M2**-(*R*, *R*))_4_ at 373 K under vacuum, which resulted in the sample becoming amorphous (Figure S27). Second, we exchanged the DEF crystallization solvent with EtOH. We then attempted to activate the EtOH-exchanged crystals of Zn_6_(**M2**-(*R*, *R*))_4_ using the more gentle supercritical CO_2_ (scCO_2_) drying technique, as used to activate low-density porous materials.^27^ Similar to Zn_6_(**M1**-(*R, R*))_4_, the PXRD patterns of Zn_6_(**M2**-(*R*, *R*))_4_ after scCO_2_ activation changed and became broad (Figure S28). However, the sample maintained some crystallinity. Subsequent thermal gravimetric analysis (TGA) also indicated that the scCO_2_ activation method was more effective in removing trace DEF solvent and water from Zn_6_(**M2**-(*R*, *R*))_4_ than the CH_3_CN solvent exchange method (Figure S29).

Next, we investigated the stability of the Zn_6_(**M1**-(*R*, *R*))_4_ and Zn_6_(**M2**-(*R*, *R*))_4_ solids by immersing solid samples of the MOPs in water and 0.1 M HCl (aq) for 10 days at RT. We then used inductively coupled plasma optical emission spectroscopy (ICP-OES) analysis to measure the Zn ion concentrations in the water and 0.1 M HCl (aq) solutions after removing the remaining solids by filtration. As shown in Table S2, the Zn_6_(**M1**-(*R*, *R*))_4_ and Zn_6_(**M2**-(*R*, *R*))_4_ solids appear to decompose in acidic solutions, based on the high concentration of Zn ions in the filtered 0.1 M HCl (aq) solutions found by ICP-OES. However, by contrast, the low (<5 ppm) Zn ion concentrations found in the filtered water solutions by ICP-OES (Table S2) indicate that the Zn_6_(**M1**-(R, R))_4_ and Zn_6_(**M2**-(R, R))_4_ solids do not decompose in water, similar to other previously reported carboxylate-based MOPs.^28^

### Permanent Porosity of Zn_6_(M1-(*R*, *R*))_4_ and Zn_6_(M2-(*R*, *R*))_4_

**Figure 5:**
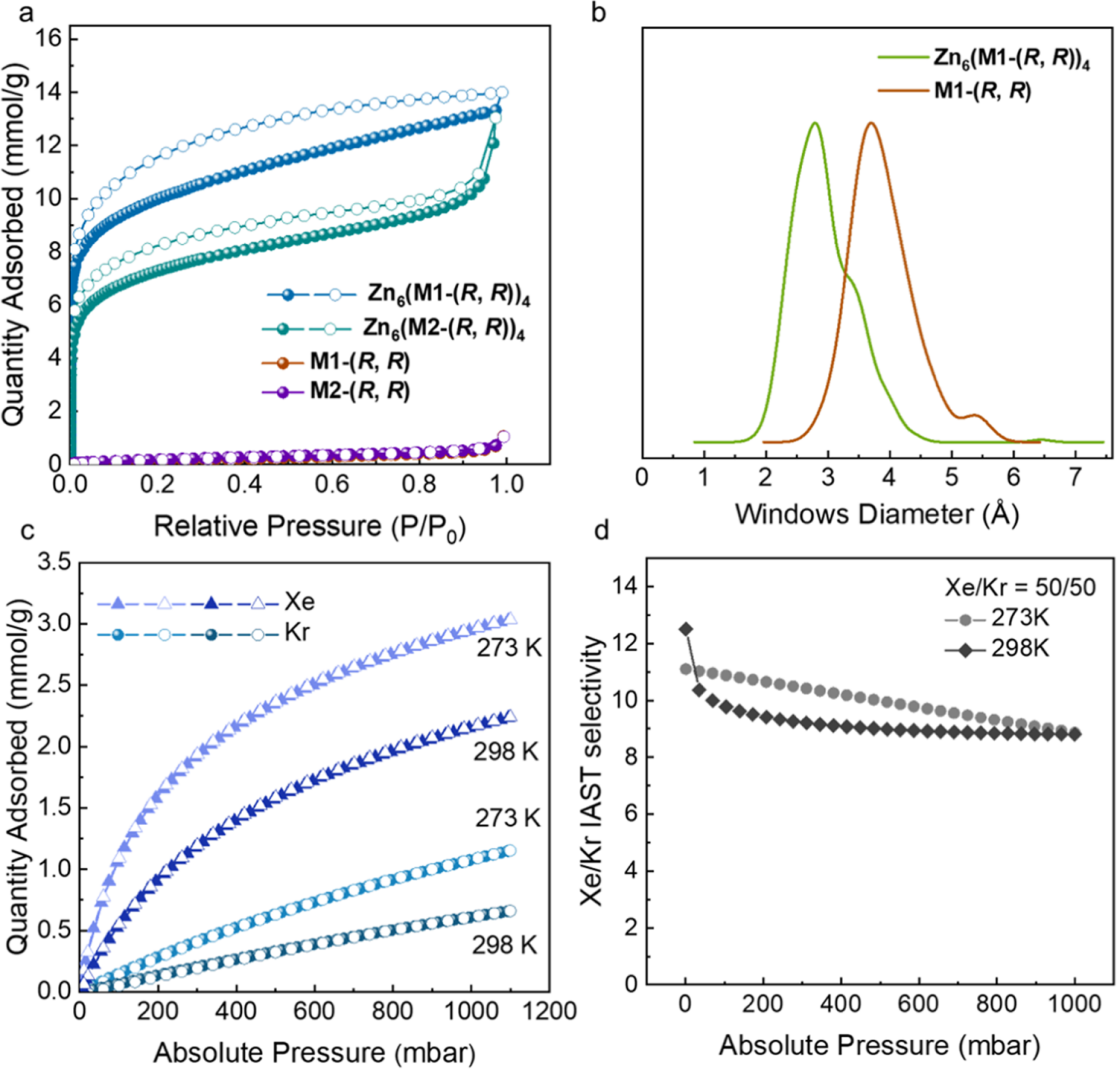
(a) N_2_ sorption isotherms recorded at 77 K. (b) Window diameter distribution histograms of **M1**-(*R*, *R*) and Zn_6_(**M1**-(*R*, *R*))_4_. (c) Xe and Kr sorption isotherms for Zn_6_(**M1**-(*R*, *R*))_4_. (d) IAST selectivity of Xe/Kr = 50/50 mixtures for Zn_6_(**M1**-(*R*, *R*))_4_ as calculated from the pure Xe and Kr gas sorption isotherms.

Although the PXRD pattern indicates that Zn_6_(**M1**-(*R, R*))_4_ and Zn_6_(**M2**-(*R, R*))_4_ lose some or all crystallinity during desolvation, this behavior is often quite common for this material class.^29^ We ran N_2_ sorption measurements to investigate the porosity of the solvent-free and activated MOPs. For these measurements, we used CH_3_CN-exchanged Zn_6_(**M1**-(*R, R*))_4_ after activation at 373 K under vacuum and the EtOH-exchanged and scCO_2_-activated Zn_6_(**M2**-(*R, R*))_4_. Both samples were degassed under vacuum before the measurements. As shown in Figure 5a, the Zn_6_(**M1**-(*R, R*))_4_ and Zn_6_(**M2**-(*R, R*))_4_ adsorption isotherms are between Type I and Type II and show pronounced uptake at low pressures (*P*/*P*_0_ < 0.05), associated with the filling of micropores in the structures. The experimental Brunauer–Emmett–Teller surface areas (*SA*_BET_) of Zn_6_(**M1**-(*R, R*))_4_ and Zn_6_(**M2**-(*R, R*))_4_ are 820 and 601 m^2^/g, respectively. By contrast, the corresponding unreacted macrocycles have far lower porosity (14 m^2^/g for **M1**-(*R, R*) and 19 m^2^/g for **M2**-(*R, R*)) based on the N_2_ isotherm shown in Figure 5a. We also recorded the gas isotherms for Zn_6_(**M2**-(*R, R*))_4_ desolvated by solvent exchange with CH_3_CN and activated at 373 K but found a lower *SA*_BET_ of 451.7 m^2^/g (Figure S31).

The predicted *SA*_BET_ values by Zeo++^30^ based on solvated single-crystal structures are 634 and 3697 m^2^/g using a 1.82 Å probe (N_2_ dynamic radius) for Zn_6_(**M1**-(*R*, *R*))_4_ and Zn_6_(**M2**-(*R*, *R*))_4_, respectively. Interestingly, the experimental *SA*_BET_ of Zn_6_(**M1**-(*R*, *R*))_4_ is higher than the predicted value based on the solvated single-crystal structure. We attribute this to Zn_6_(**M1**-(*R*, *R*))_4_ not being highly crystalline, which creates additional extrinsic porosity in the structure, and this behavior has been observed previously in less crystalline samples of other cage-based solids, such as the porous organic cage **CC3**.^31^ However, the pore size distribution (PSD) plots calculated from the N_2_ adsorption isotherm and Zeo++^32^ are similar (Figure S32), which indicates that Zn_6_(**M1**-(*R*, *R*))_4_ retains a similar structure after activation. By contrast, the experimental *SA*_BET_ of Zn_6_(**M2**-(*R*, *R*))_4_ is much lower than the calculated value, which we attribute to a combination of the MOP partially collapsing and/or the MOPs packing more densely, which is supported by the notable differences in the PSD plots calculated using the N_2_ adsorption isotherm of Zn_6_(**M1**-(*R*, *R*))_4_ and Zeo++^32^ (Figure S32).”

As shown in Figure 5b, the window diameter distribution histograms considered structurally dynamic were calculated by *py-window*(25) based on the xTB^33,34^ MD trajectory of **M1**-(*R, R*) and Zn_6_(**M1**-(*R, R*))_4_ (see Supporting Information Section 1.13). The highest probability for the window size of **M1**-(*R, R*) is around 3.7 Å, which is similar to the diameter of Kr (3.69 Å).^35^ As for Zn_6_(**M1**-(*R, R*))_4_ built from **M1**-(*R, R*), the window diameter distribution also encompasses the diameters of Kr (3.69 Å) and Xe (4.10 Å), which shows the possibility for both Kr and Xe to diffuse into the MOP cavity.^35^ In addition, the static window sizes of Zn_6_(**M1**-(*R, R*))_4_ shown in Figure 3c are 3.5 Å (for the preorganized chiral trianglsalen macrocycle type-A window) and 2.9 Å (for the type-B window). These window sizes were close to our previously studied organic cage with a static window size of 3.6 Å and the dynamic window diameter ranging from 2.9 to 4.5 Å that has been applied for separating these rare gases.^36^ Therefore, Zn_6_(**M1**-(*R, R*))_4_ has the potential for Xe/Kr adsorption and separation based on its window diameter, large intrinsic cavity around 8.7 Å, and considerable surface area. Here, we measured the single-component Xe and Kr gas isotherms for Zn_6_(**M1**-(*R, R*))_4_ at 273 and 298 K. As shown in Figure 5c, around 1100 mbar, Zn_6_(**M1**-(*R, R*))_4_ has a capacity for Xe of about 3.0 mmol/g and for Kr of about 1.2 mmol/g at 273 K, which are close to the performance of the porous organic cage CC3 for these two gases (Xe = 2.5 mmol/g vs Kr = 1.5 mmol/g at 273 K).^36^ In addition, the uptake for Xe at 298 K and 1 bar in Zn_6_(**M1**-(*R, R*))_4_ (2.15 mmol/g) is comparable to MOF-5 (1.98 mmol/g) that has a *SA*_BET_ around 3400 m^2^/g, HOF-BTB (2.1 mmol/g) that has a *SA*_BET_ of 1050 m^2^/g, and MOP 1-Zn (1.98 mmol/g) that has a *SA*_BET_ of 416 m^2^/g.^37−39^

The Xe–Kr binary mixture adsorption selectivity was then predicted using ideal adsorption solution theory (IAST) based on the single-gas isotherms shown in Figure 5c.^40^ The IAST-predicted selectivity is shown in Figure 5d for binary mixtures of Xe–Kr with compositions of 50:50 at 273 and 298 K. For equimolar mixtures, the predicted selectivity was 12.5 at very low pressure and then gradually decreased to 8.8 at 1000 mbar and 298 K. At 273 K, for Xe–Kr with compositions of 50:50, the predicted selectivity from 11.1 to 8.9 was higher than that of HOF-BTB (around 3–6.8), which has been used previously for the selective separation of Xe from Kr in breakthrough experiments.^38^ As such, the MOP studied in this work has potential for rare gas separation, perhaps after being processed into a membrane similarly to other types of porous cages,^41^ since the MOP has good solubility in organic solvents and, therefore, good processability.

## Conclusions

In summary, two chirally pure, octahedral MOPs, Zn_6_(**M1**-(*R, R*))_4_ and Zn_6_(**M2**-(*R, R*))_4_, were synthesized via the self-assembly of trianglsalen macrocycles and Zn(II) cations. The structures of Zn_6_(**M1**-(*R, R*))_4_ and Zn_6_(**M2**-(*R, R*))_4_ were confirmed by SC-XRD, NMR, and mass spectrometry, which revealed that four trianglsalen macrocycles are triply deprotonated and linked together by six Zn(II) cations in each MOP. The MOPs have eight windows, including four windows from the used trianglsalen macrocycles and four windows from the connection of three neighboring macrocycles with three Zn(II) cations. The MOPs inherit and amplify the chirality from the chiral macrocycles and can resist degradation in water. By extending the length of the dialdehydes, we increase the cavity of macrocycles from 4.2 to 6.5 Å, which systematically increases the MOP window dimensions from 3.5 to 5.9 Å for type-A windows and from 2.9 to 4.5 Å for type-B windows, concurrently with increasing the MOP cavity size from 26.3 to 32.1 Å. Compared with the nonporous trianglsalen macrocycles, Zn_6_(**M1**-(*R, R*))_4_ and Zn_6_(**M2**-(*R, R*))_4_ exhibit experimental *SA*_BET_ values of 820.1 and 601.3 m^2^/g, respectively. Zn_6_(**M1**-(*R, R*))_4_ exhibits capacities for Xe and Kr of about 3.0 and about 1.2 mmol/g, respectively, at 273 K and 1100 mbar, and the IAST selectivity for Xe/Kr ranges from 11.1 to 8.9 at 273 K. This work provides a new strategy for integrating macrocycles as intrinsically porous ligands in the assembly of MOPs that then inherit the structural features of the building blocks, such as chiral amplification and host–guest chemistry.

## Supporting Information Available

The Supporting Information is available free of charge at Figures S1–S33, Tables S1–S2. Experimental details and synthetic procedures; NMR spectra; MALDI-TOF spectra; PXRD and variable-temperature PXRD patterns; SC-XRD refinement details and crystal structure images; TGA plots; and gas sorption analysis data (PDF)
