# Supplementary material for: Self-Assembly of Chiral Porous Metal–Organic Polyhedra from Trianglsalen Macrocycles
Source: J Am Chem Soc. 2024 Jun 11;146(25):17438–45. doi: 10.1021/jacs.4c04928 (PMC11212058; doi:10.1021/jacs.4c04928)
Supplement: Supplementary file 1 — ja4c04928_si_001.pdf [file ja4c04928_si_001.pdf]

Supplementary Information:

Self-assembly of Chiral Porous Metal-organic Polyhedra from Trianglsalen Macrocycles

Donglin He, Heng Ji, Tao Liu, Miao Yang, Rob Clowes, Marc A. Little,* Ming Liu,* and Andrew I. Cooper*

1. Experimental Details

1.1. Materials

(1*R*,2*R*)-Cyclohexane-1,2-diamine was purchased from Manchester Organics, UK. 3,3'-Dihydroxy[1,1'-biphenyl]-4,4'-dicarboxaldehyde was purchased from Shanghai Sunway Corporation Ltd. All other chemicals were purchased from Sigma-Aldrich and used as received.

1.2. NMR Spectra

NMR spectra were recorded at ambient probe temperature on a Bruker 400 NMR spectrometer at 400 MHz (^{1}H) and 100 MHz (^{13}C) and referenced against the residual ^{1}H or ^{13}C signal of the solvent.

1.3. Infra-red (IR) Spectra

IR spectra were recorded on a Bruker Tensor 27 FT-IR using ATR measurements for solids as neat samples, or using transmission mode on a 96-well silica wafer deposited as a thin film as part of the high-throughput analysis.

1.4. Elemental analysis

CHN analysis was performed on a Thermo EA1112 Flash CHNS-O Analyser using standard microanalytical procedures.

1.5. High resolution mass spectrometry (HRMS)

HRMS was carried out using an Agilent Technologies 6530B accurate-mass QTOF Dual ESI mass spectrometer (capillary voltage 4000 V, fragmentor 225 V) in positive-ion detection mode. The mobile phase was methanol (MeOH) + 0.1% formic acid at a flow rate of 0.25 mL/min.

1.6. Matrix-assisted laser desorption/ionisation time of flight mass spectrometry (MALDI-TOF MS)

MALDI-TOF MS was conducted using an AXIMA Confidence MALDI MS (Shimadzu Biotech) fitted with a 50 Hz N_2 laser. A 10:1 -5-1 ratio of matrix/sample was dissolved in tetrahydrofuran (THF, 10 mg mL^{-1}) and this was drop-coated onto the microtitre plate before analysis. The matrix used was *trans*-2-[3-(4-*tert*-butylphenyl)-2-methyl-2-propenylidene]malononitrile (DCTB).

1.7. Powder X-ray diffraction

Laboratory powder X-ray diffraction (PXRD) data patterns were collected in transmission mode on samples held on thin Mylar film in aluminium well plates on a Panalytical Empyrean diffractometer, equipped with a high throughput screening XYZ stage, X-ray focusing mirror, and PIXcel detector, using Cu-Kα (λ = 1.541 Å) radiation. For indexing, samples were loaded into borosilicate glass capillaries, and PXRD patterns were recorded in transmission mode on a Panalytical Empyrean diffractometer, equipped with a sample spinner, X-ray focusing mirror, and PIXcel detector, using Cu-Kα (λ = 1.541 Å) radiation.

1.8. Single crystal X-ray diffraction (SC-XRD)

SC-XRD data sets were measured on a Rigaku MicroMax-007 HF rotating anode diffractometer (Mo-Kα radiation, λ = 0.71073 Å, Kappa 4-circle goniometer, Rigaku HyPix-6000HE hybrid pixel array detector). Absorption corrections, using the multi-scan method, were performed with the program CrysAlisPro 1.171.40.45a. For details on the full refinement, see Table S1 and the supporting CIFs.

1.9. Gas sorption

Nitrogen isotherms of **M1** and **M2** were collected at 77 K using an ASAP2020 volumetric adsorption analyzer (Micrometrics Instrument Corporation). **M1** and **M2** were degassed at 100 °C for 15 hours under a dynamic vacuum prior to gas analysis. Isotherm measurements of MOPs were performed using a Micromeritics 3flex surface characterization analyzer, equipped with a Cold-Edge technologies liquid helium cryostat chiller unit for temperature control. N_2 isotherms for MOPs were collected at 77 K. Kr and Xe isotherms for MOPs were collected at 273 and 298 K. MOPs activated by solvent exchange method using acetonitrile (MeCN) were degassed at 100 °C for 15 hours under dynamic vacuum prior to gas analysis. Zn_6(**M2**-(*R*, *R*))$_4$ activated by the supercritical CO_2 drying was degassed at room temperature for 15 hours under dynamic vacuum prior to gas analysis.

1.10. Thermogravimetric analysis (TGA)

TGA was carried out using a Q5000IR analyzer (TA instruments) with an automated vertical overhead thermobalance. The samples were heated at the rate of 10 °C /min using dry N_2 as the protective gas.

1.11. CD spectroscopy

Appropriate amounts of dried **M1**-(*S*, *S*), **M1**-(*R*, *R*), **M2**-(*S*, *S*), **M2**-(*R*, *R*), Zn_6(**M1**-(*S*, *S*))$_4$ and Zn_6(**M1**-(*R*, *R*))$_4$ were weighed into sample bottles and dissolved into DCM. Then the solutions were diluted to the solutions with the suitable concentration. The final concentrations of **M2**-(*R*, *R*) and Zn_6(**M1**-(*R*, *R*))$_4$ for the test are 0.10 mg/mL. As for the rest solutions, the final concentrations are 0.25 mg/mL. As for Zn_6(**M2**-(*S*, *S*))$_4$ and Zn_6(**M2**-(*R*, *R*))$_4$, the samples as synthesized in DEF were directly dissolved into DCM. The concentration of Zn_6(**M2**-(*S*, *S*))$_4$ and Zn_6(**M2**-(*R*, *R*))$_4$ were estimated by the yield calculated by the three same parallel reactions. The final detection concentrations of Zn_6(**M2**-(*S*, *S*))$_4$ and Zn_6(**M2**-(*R*, *R*))$_4$ after further dilution were 0.60 mg/mL and 0.35 mg/mL, respectively. Then, the CD (mdeg) signal of each sample was determined by a circular dichroism spectrometer (model: JASCO J-1700, testing optical path: 1 mm). The mdeg was converted to molar ellipticity by calculation. The formula is $[\theta](deg\cdot cm^2\cdot dmol^{-2}) = mdeg\cdot 10^3/(l\cdot c)$ (l (mm) represents the optical path, and c (mM) represents the molar concentration of the sample).

1.12 Inductively coupled plasma optical emission spectrometry (ICP-OES)

ICP-OES measurements were conducted on an ICP-OES Agilent 5110. Before the measurements, the ICP-OES instrument was calibrated using four standards at three different wavelengths (202.548 nm, 213.857 nm, and 472.215 nm), and the relative standard deviations (% RSDs) were determined from the calibration measurements. The filtered MOPs immersing solutions were diluted using distilled water before analysis.

1.13. Computational details

An isolated molecule, extracted from the experimental crystal structure of **M1**-(*R*, *R*) and Zn_6(**M1**- (*R*, *R*))$_4$, was optimized by the GFN2-XTB method with D4 dispersion[1] model in the gas phase with defaults for convergence. The optimized geometry was confirmed as a true minima by numerical harmonic frequency calculation without imaginary frequency. [2-4] Based on that, MD simulation was performed for 200 ps, in which 100 ps for equilibration and 100 ps for production with timestep of 2 fs, along with SHAKE restraints on all bonds,[5] in the NVT ensemble using the Berendsen thermostat[6] to maintain the temperature of 298 K. Structures were dumped every 2 ps.

Pywindow[7] was used to calculate the widows diameter of the molecular dynamics trajectories including 50 geometries obtained from xTB calculations.

2. Synthetic procedures

All enantiomers were synthesized using the same reaction procedures by using the appropriate chiral start materials.

2.1 Synthesis of macrocycle M1

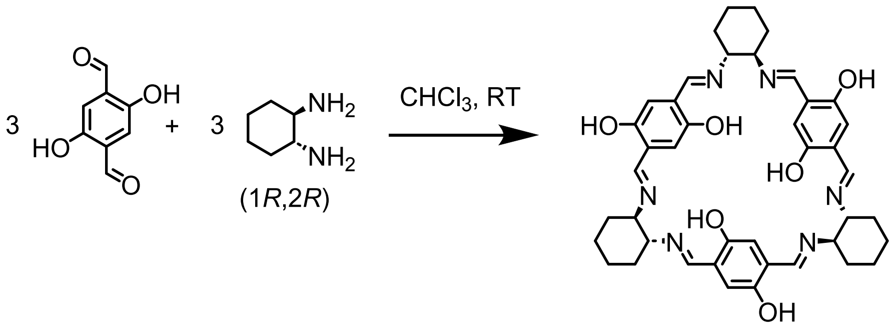

M1-(*R*, *R*)

Figure S1. Scheme for the synthesis of **M1**-(*R*, *R*).

M1-(*R*, *R*) was synthesized as described previously[8] with the slight adjustments to the method. A solution of trans-(1*R*,2*R*)-diaminocyclohexane (560 mg, 5 mmol) in 100 ml was added into 150 ml solution of 2,5- dihydroxyterephthalaldehyde (815 mg, 5 mmol) in $CHCl_3$. The mixture was stirred under nitrogen atmosphere at room temperature for 7 days. After that time, to the mixture was added ethanol (100 mL). The product crystallized as yellow-orange solid in 80% yield.

^{1}H NMR (400 MHz, $CDCl_3$) δ_H 12.26 (6H, s, O*H*), 8.16 (6H, s, N=C*H*), 6.68 (6H, s, Ar*H*), 3.31 - 3.28 (6H, m, C*H*-N), 1.71 - 1.43 (24H, m, cyclohexyl CH_2). ^{13}C NMR (101 MHz, $CDCl_3$) δ = 163.92, 152.50, 121.01, 118.38, 73.83, 32.97, 24.19. HRMS: [$C_{42}H_{48}N_6O_6$] calcd at 732.3635, $[M+H]^+$, m/z found 733.3718. Elemental Analysis: C, 67.86; H 6.60; N 11.40 (C, 68.83; H 6.60; N 11.47 calculated for $C_{42}H_{48}N_6O_6$). IR (V_{max}/ cm^{-1}): 770.58, 785.85, 800.15, 854.13, 1038.99, 1093.06, 1155.29, 1215.06, 1309.79, 1346.58, 1448.06, 1505.05, 1621.68, 2858.13, 2923.82. (See Figure S2 – 3 for NMR spectra, Figure S4 for MS and Figure S17 for FT-IR spectra) Data in accordance with literature values.[8]

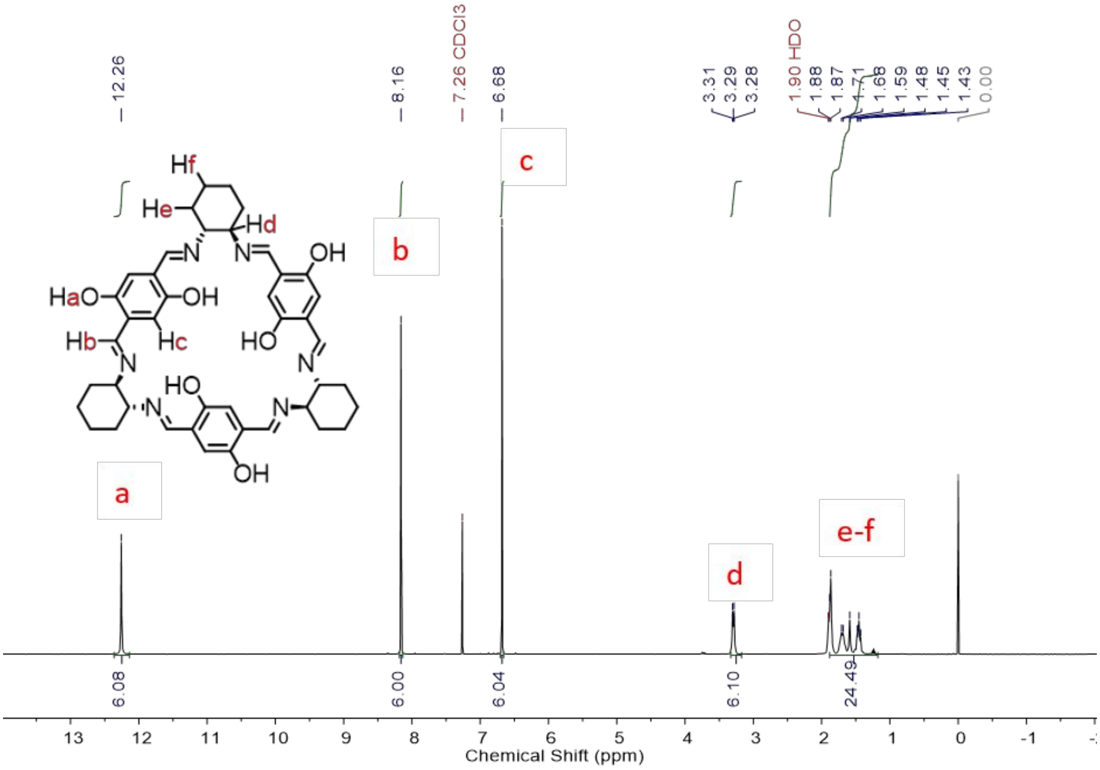

Figure S2. ^{1}H NMR spectrum (400 MHz, $CDCl_3$, 293 K) of **M1**-(*R*, *R*).

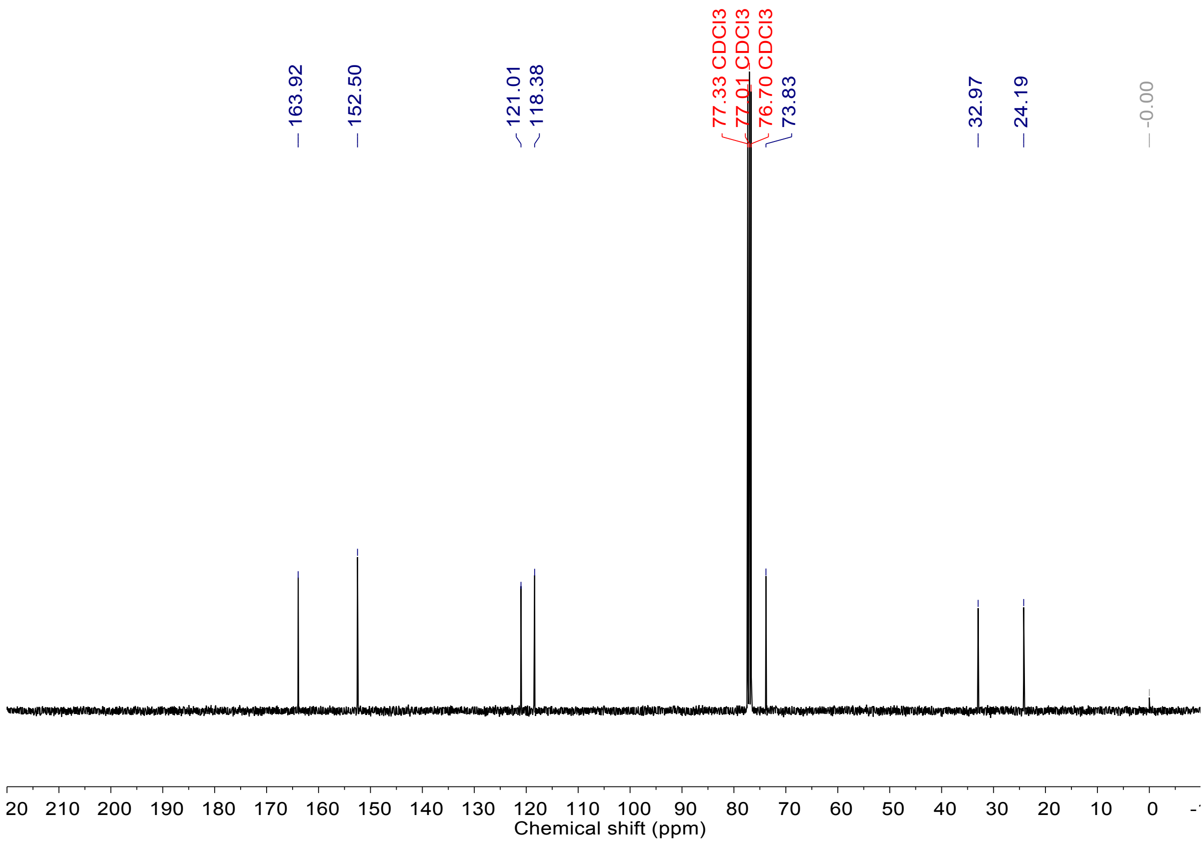

Figure S3. ^{13}C NMR spectrum (101 MHz, $CDCl_3$, 293 K) of **M1**-(*R*, *R*).

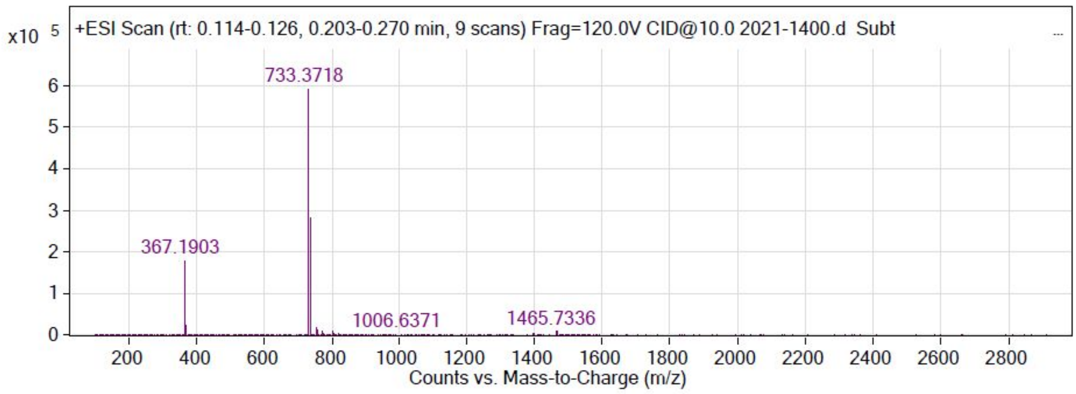

Figure S4. MS $[M+H]^+$ of **M1**-(*R*, *R*).

2.2 Synthesis of macrocycle M2

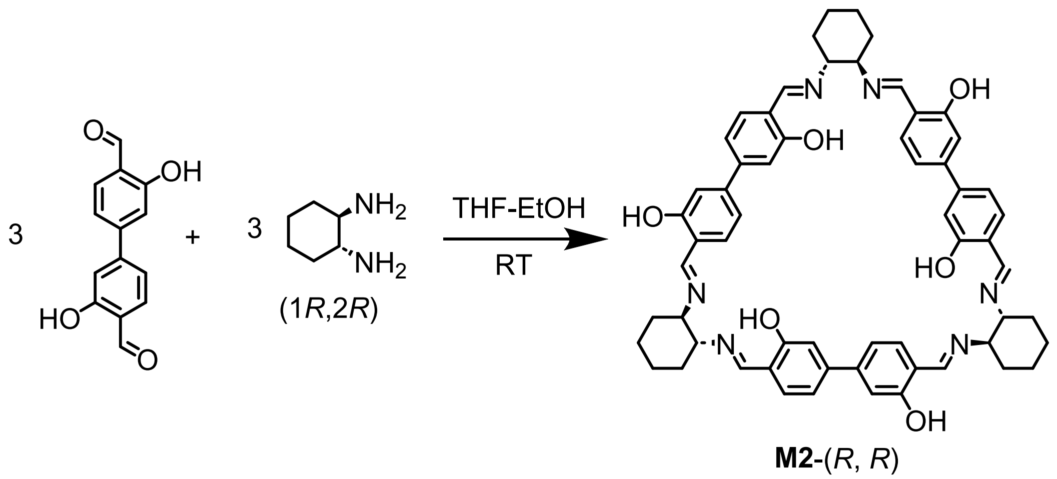

Figure S5. Scheme for the synthesis of **M2**-(*R*, *R*).

3,3'-Dihydroxy[1,1'-biphenyl]-4,4'-dicarboxaldehyde (121 mg 0.5 mmol) was dissolved in tetrahydrofuran (25 mL). Then, 25 mL (1*R*,2*R*)-1,2-diaminocyclohexane (57 mg, 0.5 mmol) dissolved in ethanol added to the reaction slowly. The mixture was stirred at room temperature for 12 h, then the solvent was evaporated at room temperature. The yellow solid product was obtained by filtering and washing with ethanol. Yield: 107.6 mg (67.3%).

^{1}H NMR (400 MHz, $CDCl_3$) δ_H 13.27 (6H, s, O*H*), 8.22 (6H, s, N=C*H*), 7.14 (6H, d, *J* = 8 Hz, N=C*H*), 7.06 (6H, s, Ar*H*), 6.96 (6H, d, *J*= 4 Hz, *J*= 8, Ar*H*), 3.27-3.49 (6H, m, C*H*-N), 1.48-1.77 (24H, m, cyclohexyl CH_2). ^{13}C NMR (400 MHz, $CDCl_3$) δ_C 164.44, 160.96, 143.69, 131.78, 117.92, 117.65, 114.95, 73.06, 32.82, 24.29. HRMS: [$C_{60}H_{60}N_6O_6$] calcd at: 960.4574. Found $[M+H]^+$ at 961.4654. Elemental Analysis: C, 73.64; H 6.29; N 8.62 (C, 74.98; H 6.29; N 8.74 calculated for $C_{60}H_{60}N_6O_6$). IR (V_{max}/ cm^{-1}): 794.85, 883.81, 1146.11, 1202.85, 1250.99, 1357.79, 1550.13, 1619.75, 2855.22, 2926.02. (See Figure S6 - 7 for NMR spectra, Figure S8 for MS and Figure S21 for FT-IR spectra)

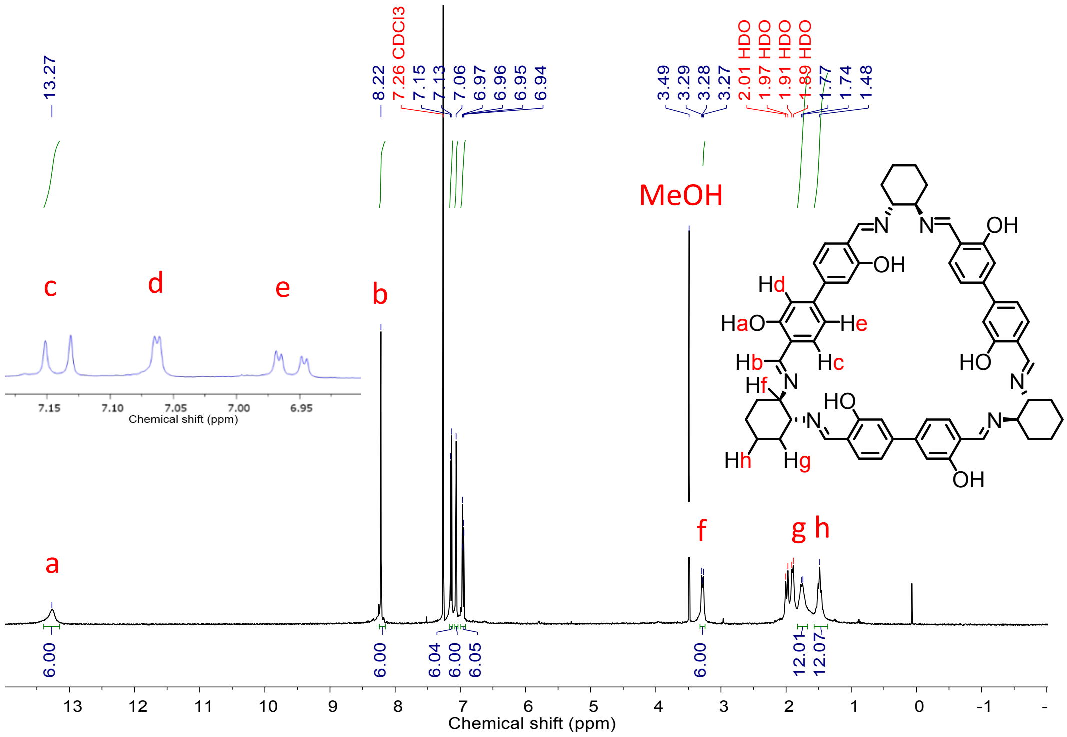

Figure S6. ^{1}H NMR spectrum (400 MHz, $CDCl_3$, 293 K) of **M2**-(*R*, *R*).

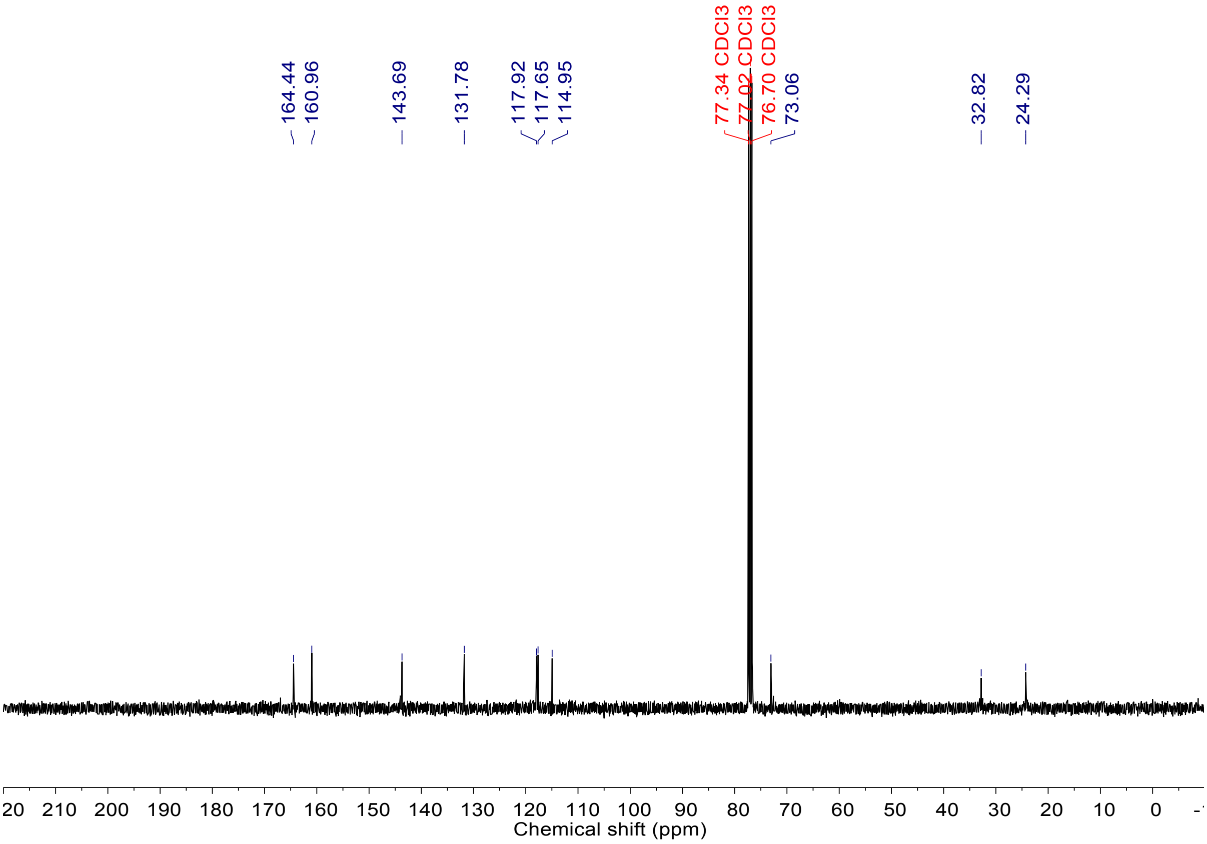

Figure S7. ^{13}C NMR spectrum (101 MHz, $CDCl_3$, 293 K) of **M2**-(*R*, *R*).

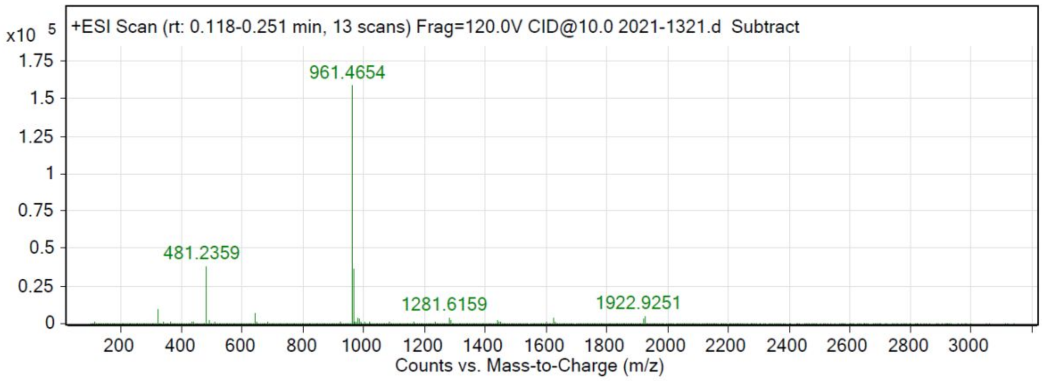

Figure S8. MS $[M+H]^+$ of **M2**-(*R*, *R*).

2.3 Synthesis of macrocycle M3

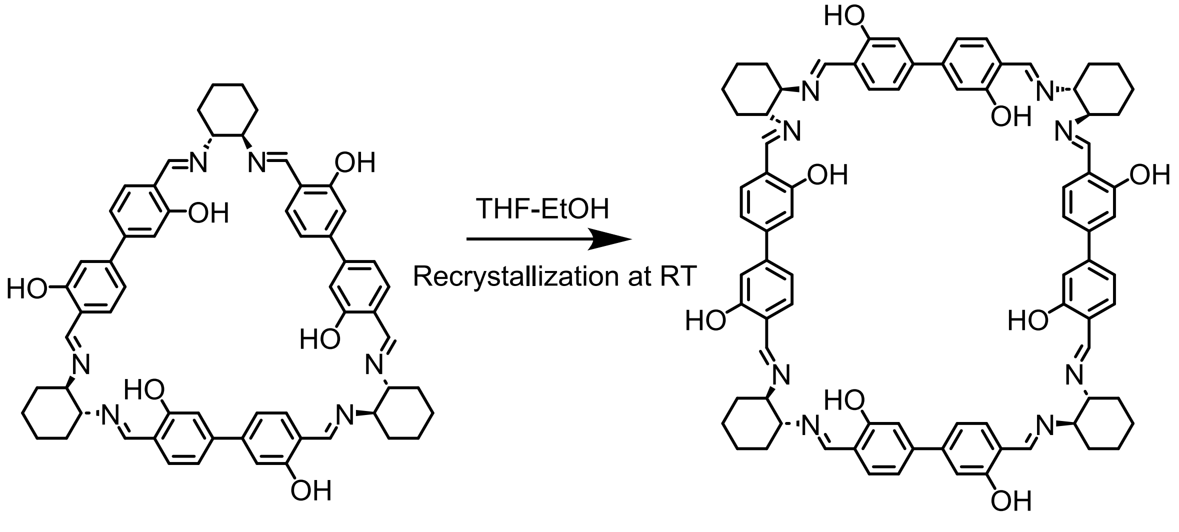

M2-(*R*, *R*)　　　　　　　　**M3**-(*R*, *R*)

Figure S9. Scheme for the synthesis of **M3**-(*R*, *R*).

M2 (50 mg) was dissolved in tetrahydrofuran (10 mL). After filtering the mixture through a syringe filter (0.2 µm PTFE membrane), the clear solution was divided evenly into five vials (2 mL per vial). Ethanol was allowed to vapour-diffuse slowly into the vials over a period of 3 to 7 days at room temperature. Yellow needle crystals of **M3**-(*R*, *R*) were obtained by filtering and washing with ethanol. Yield: 23 mg (46.0%).

^{1}H NMR (400 MHz, $CDCl_3$) δ_H 13.40 (6H, s, O*H*), 8.25 (6H, s, N=C*H*), 7.16 (6H, d, *J* = 8 Hz, N=C*H*), 7.07 (6H, s, Ar*H*), 6.98 (6H, d, *J*= 4 Hz, *J*= 8, Ar*H*), 3.30-3.33 (6H, m, C*H*-N), 1.45-1.98 (24H, m, cyclohexyl CH_2). ^{13}C NMR (400 MHz, $CDCl_3$) δ_C 164.36, 161.10, 144.14, 131.80, 118.04, 117.68, 115.41, 72.61, 33.14, 24.27. HRMS: [$C_{80}H_{80}N_8O_8$] calcd at: 1280.6099. Found $[M+H]^+$ at 1281.6141. Elemental Analysis: C, 73.89; H 6.35; N 8.65 (C, 74.98; H 6.29; N 8.74 calculated for $C_{80}H_{80}N_8O_8$). IR (V_{max}/ cm^{-1}): 794.99, 882.70, 1141.18, 1201.11, 1252.52, 1356.62, 1549.42, 1619.46, 2853.99, 2925.77. (See Figure S10- 11 for NMR spectra, Figure S12 for MS spectra)

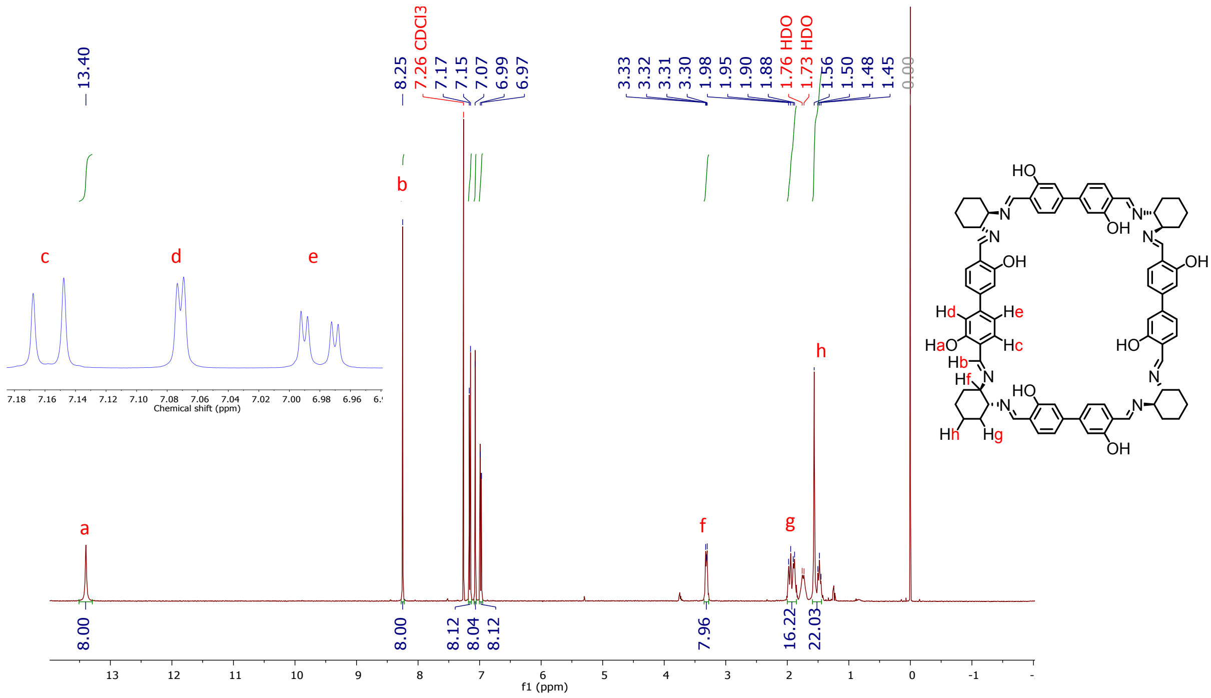

Figure S10. ^{1}H NMR spectrum (400 MHz, $CDCl_3$, 293 K) of **M3**-(*R*, *R*).

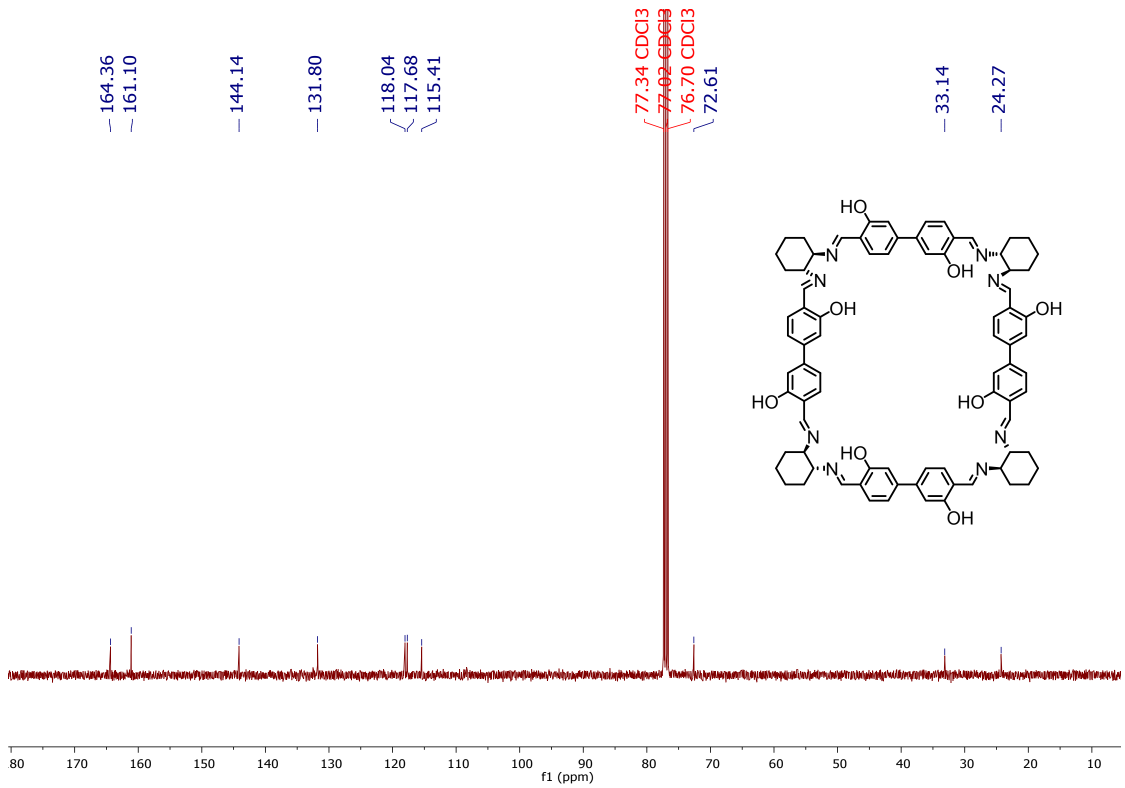

Figure S11. ^{13}C NMR spectrum (101 MHz, $CDCl_3$, 293 K) of **M3**-(*R*, *R*).

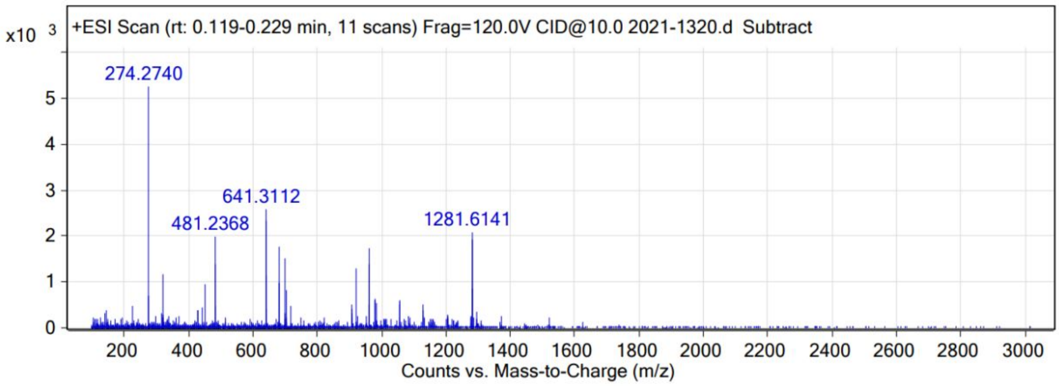

Figure S12. MS $[M+H]^+$ of **M3**-(*R*, *R*).

2.4 Synthesis of $Zn_6(M1)_4$

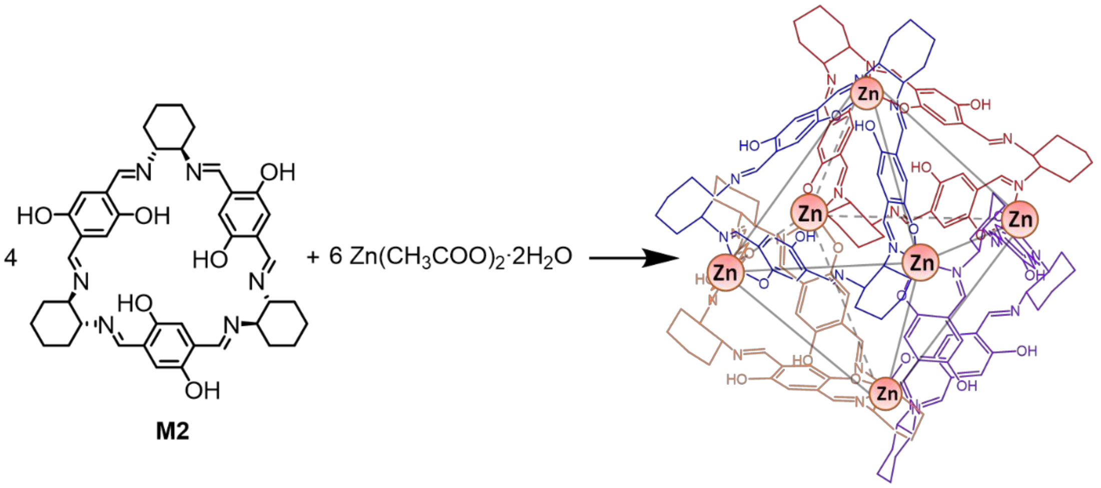

Figure S13. Scheme for the synthesis of Zn_6(**M1**-(*R*, *R*))$_4$.

A solution of 66 mg (0.30 mmol) $Zn(CH_3COO)_2{\cdot}2H_2O$ dissolved in DEF (15 mL) was added to the stirred solution of the 3+3 macrocycle **M1**-(*R*, *R*) (146 mg, 0.20 mmol) in DEF (15 mL). The mixed solution was then put into a small vial. The small vial was loaded into a larger vial containing CH_3CN, and capped. After 7 days, orange crystal had formed in the small glass vial and the residual solvent was removed using a syringe. CH_3CN was added to fully immerse the crystals and the CH_3CN solvent was exchanged again every 24 hours for 5 days. After that, the desolvated **Zn_6(M1**-(*R*, *R*)**)$_4$** (105 mg, 64.2 %) was obtained by filtering and drying at 100 °C in vacuum for 12 h.

^{1}H NMR (400 MHz, $CDCl_3$) δ_H 11.62 (3H, s, O*H*), 8.36 (3H, s,N=C*H*), 8.10 (3H, s,N=C*H*), 6.78 (3H, s, Ar*H*), 6.55 (3H, s, Ar*H*), 3.71 (3H, m, C*H*-N), 3.46 (3H, m, C*H*-N),1.35-1.78 (24H, m, cyclohexyl C*H*$_2$); δ_C 170.63, 167.13, 163.92, 148.26, 126.25, 126.13, 120.29, 119.14, 76.78, 71.25, 24.47; MALDI-TOF MS: [$C_{168}H_{180}N_{24}O_{24}Zn_6$] calcd at 3311.712. Found $[M+H]^+$ at 3312.146. Elemental Analysis: C, 58.00; H, 5.46; N, 9.59 (C, 60.93; H 5.48; N 10.15 calculated for $C_{168}H_{180}N_{24}O_{24}Zn_6$). IR ($V_{max}$/ cm^{-1}): 818.72, 865.27, 1135.53, 1153.40, 1218.24, 1311.03, 1371.01, 1471.97, 1603.01, 2856.88, 2927.36. (See Figure S14-15 for NMR spectra, Figure S16 for MS and Figure S17 for FT-IR spectra)

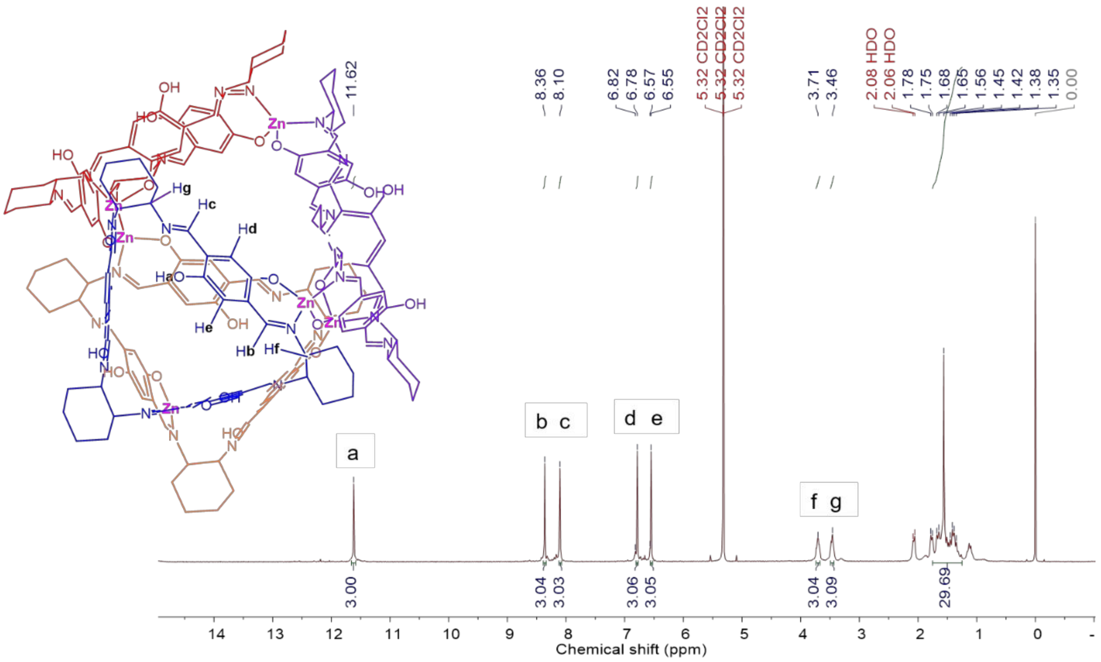

Figure S14. ^{1}H NMR spectrum (400 MHz, $CDCl_3$, 293 K) of Zn_6(**M1**-(*R*, *R*))$_4$.

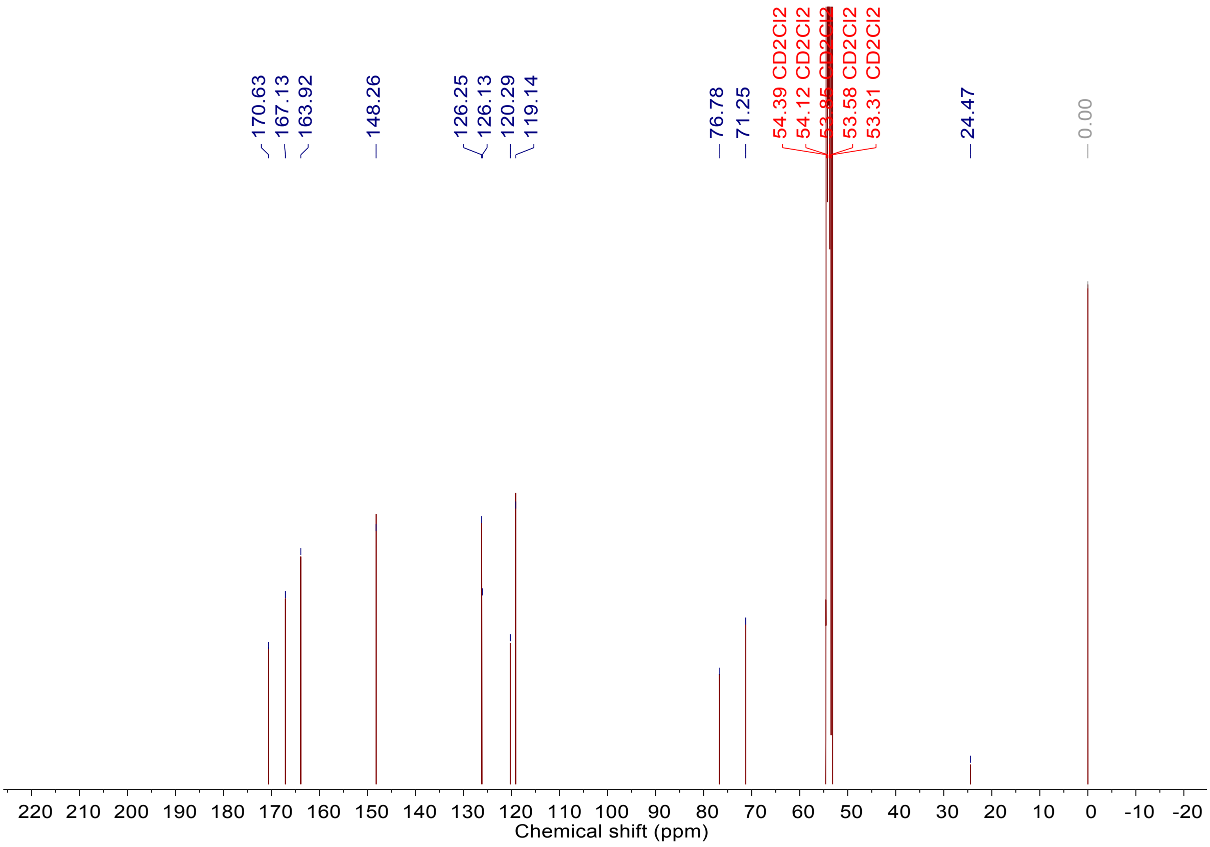

Figure S15. ^{13}C NMR spectrum (101 MHz, CD_2Cl_2, 293 K) of Zn_6(**M1**-(*R*, *R*))$_4$.

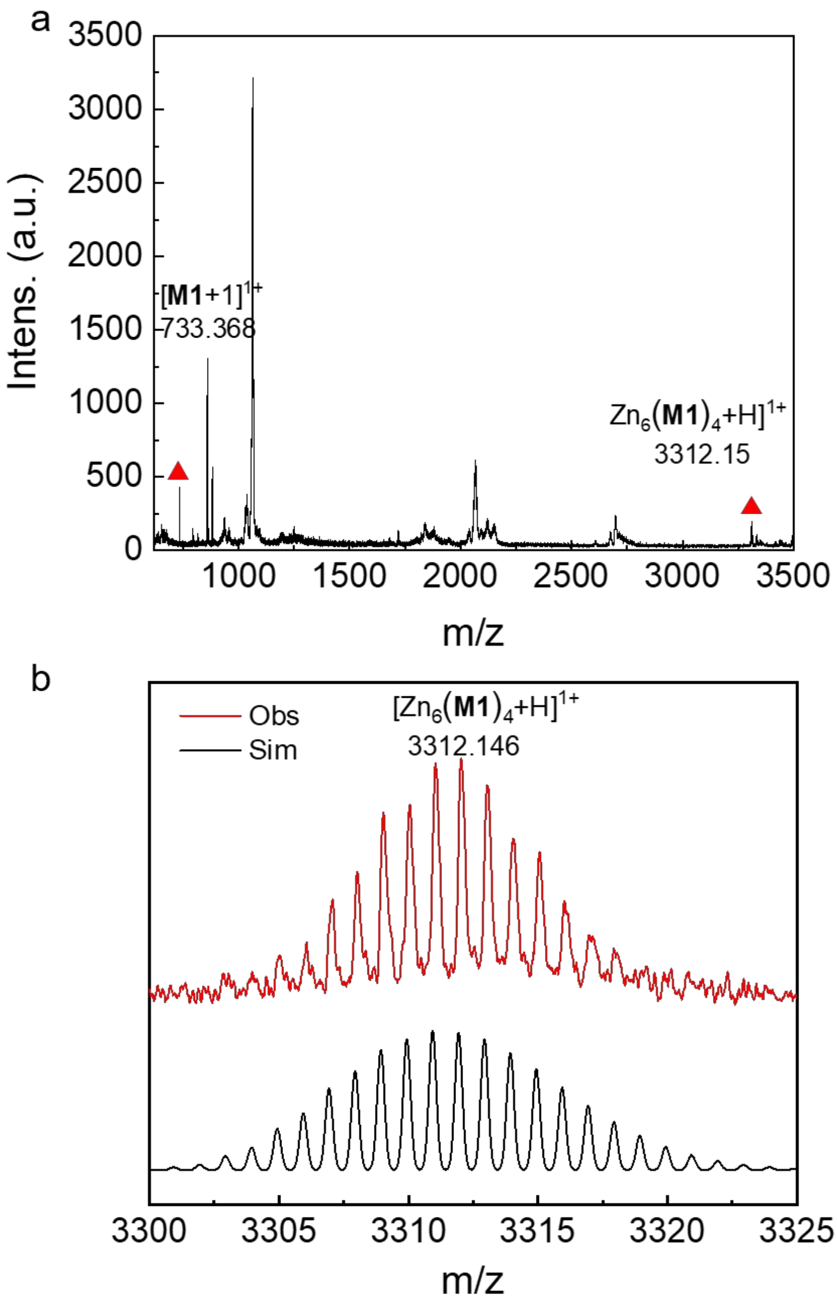

Figure S16. MALDI-TOF MS $[M+H]^+$ of macrocycle of Zn_6(**M1**-(*R*, *R*)$)_4$: (a) whole spectrum and (b) partial enlarged spectrum.

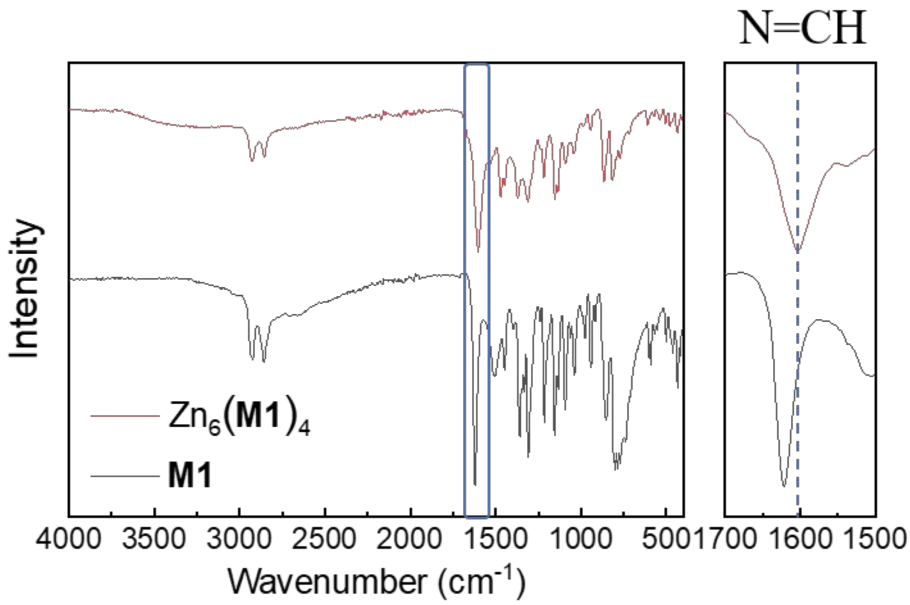

Figure S17. FT-IR spectra of **M1**-(*R*, *R*) and Zn_6(**M1**-(*R*, *R*))$_4$.

2.5 Synthesis of $Zn_6(M2)_4$

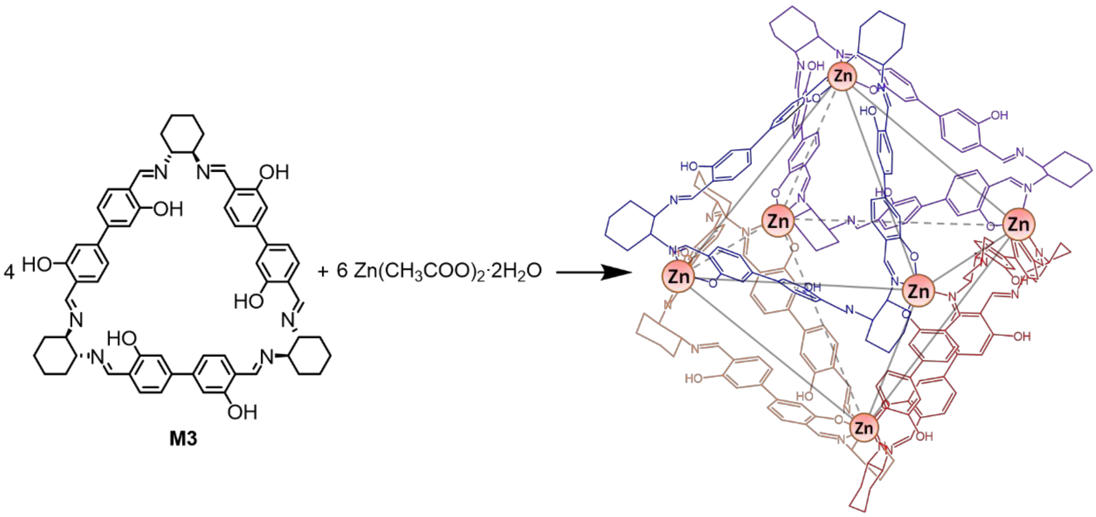

Figure S18. Scheme for the synthesis of Zn_6(**M2**-(*R*, *R*))$_4$.

A solution of 27 mg (0.012 mmol) $Zn(CH_3COO)_2{\cdot}2H_2O$ dissolved in DEF (20 mL) was added to the stirred solution of the 3+3 macrocycle **M2**-(*R*, *R*) (78 mg, 0.08 mmol) in DEF (40 mL). The mixture was stirred at room temperature for 10 min. Then the solution was filtered, put into a flask and left to stand at room temperature. After 3 days, yellow block crystals had formed in the flask, and the residual solvent was removed using a syringe. CH_3CN was added to immerse the crystals fully, and the CH_3CN solvent was exchanged every 24 hours for 5 days. After that, the desolvated Zn_6(**M2**-(*R*, *R*))$_4$ (53 mg, 62.7%) was obtained by filtering and drying at 100 °C under a vacuum for 12 h.

^{1}H NMR (400 MHz, $CDCl_3$) δ_H 13.59 (3H, , s, O*H*), 8.66 (3H, s,N=C*H*), 8.15 (3H, s,N=C*H*), 7.35 (3H, d, *J* = 8 Hz, Ar*H*), 7.13 (3H, d, *J* = 8 Hz, Ar*H*), 7.11 (3H, s, Ar*H*), 7.09 (3H, s, Ar*H*), 7.03 (3H, s, Ar*H*), 6.74 (3H, dd, *J*= 8 Hz, *J*= 8, Ar*H*), 3.46 (3H, m, C*H*-N), 2.20 (3H, m, C*H*-N), 1.41-1.73 (24H, m, cyclohexyl C*H*$_2$); MALDI-TOF MS: [$C_{240}H_{228}N_{24}O_{24}Zn_6$] calculated at 4224.888. Found $[M+2H]^{2+}$ at 2115.380, $[M+2H+2Zn]^{2+}$ at 2179.570. Elemental Analysis: C, 67.13; H, 5.39; N, 7.85 (C, 68.23; H 5.44; N 7.96 calculated for $C_{240}H_{228}N_{24}O_{24}Zn_6$). IR ($V_{max}$/ cm^{-1}): 788.99, 865.28, 888.86, 1190.18, 1371.47, 1506.62, 1594.57, 2855.66, 2925.64. (See Figure S19 for NMR spectra, Figure S20 for MS and Figure S21 for FT-IR spectra)

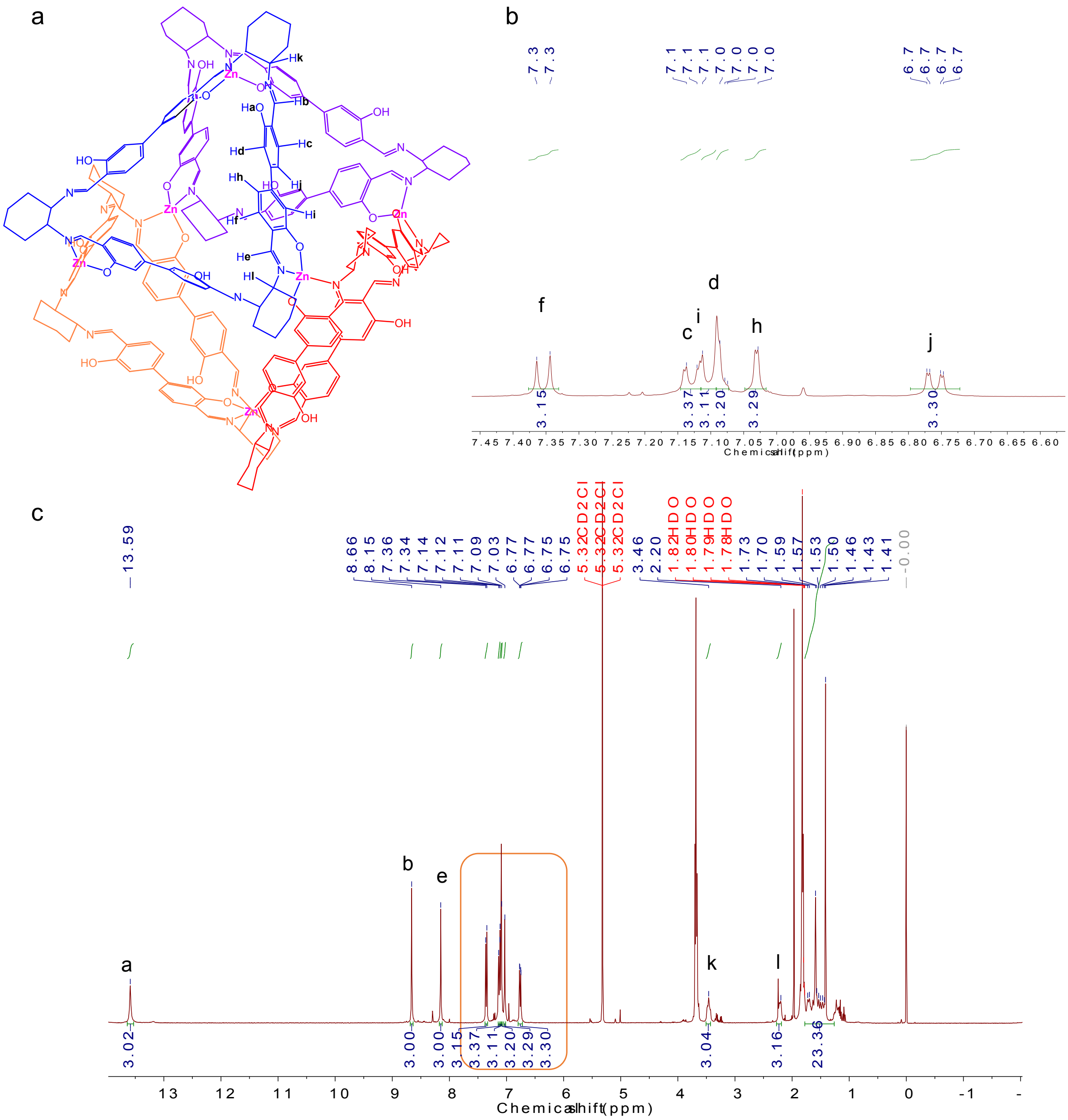

Figure S19. ^{1}H NMR spectrum (400 MHz, $CDCl_3$, 293 K) of Zn_6(**M2**-(*R*, *R*))$_4$.

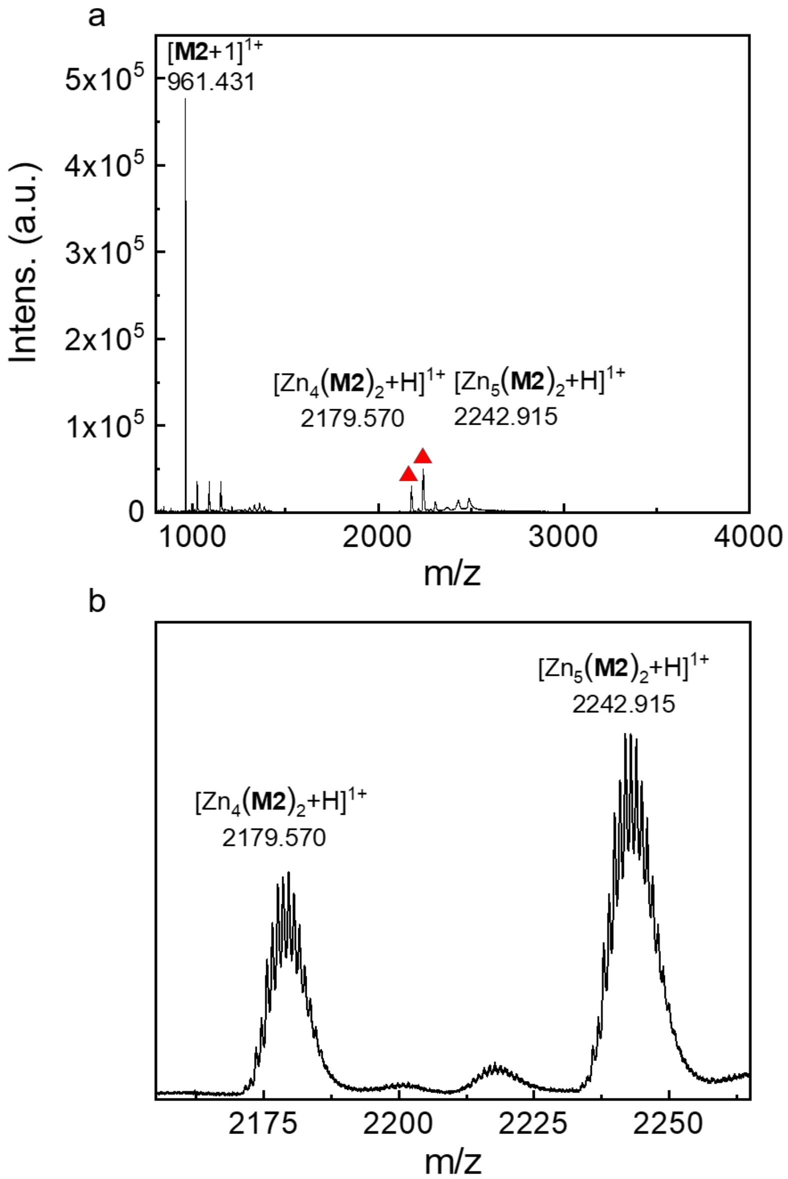

Figure S20. MALDI-TOF MS $[M+H]^+$ of Zn_6(**M2**-(*R*, *R*))$_4$: (a) whole spectrum and (b) partial enlarged spectrum.

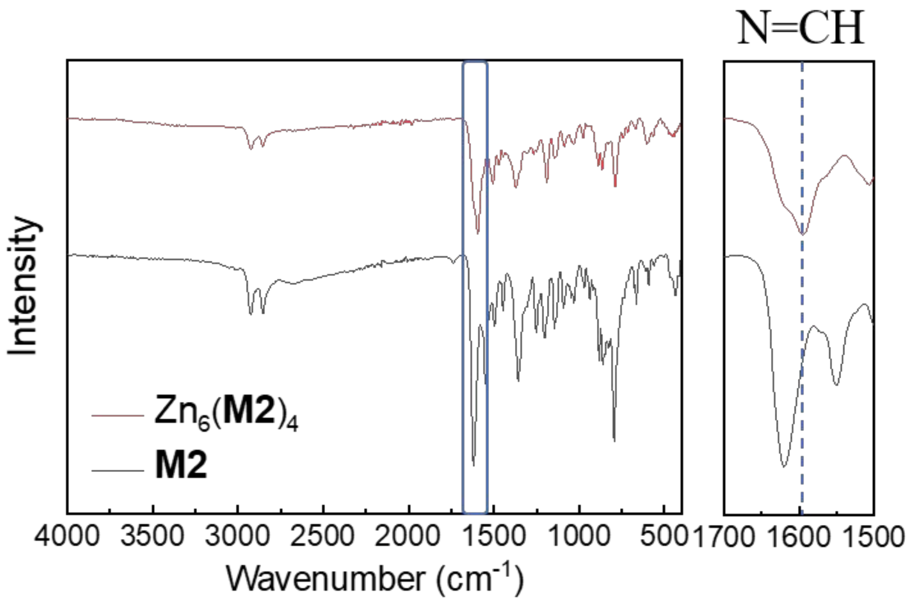

Figure S21. FT-IR spectra of **M2**-(*R*, *R*) and Zn_6(**M2**-(*R*, *R*))$_4$.

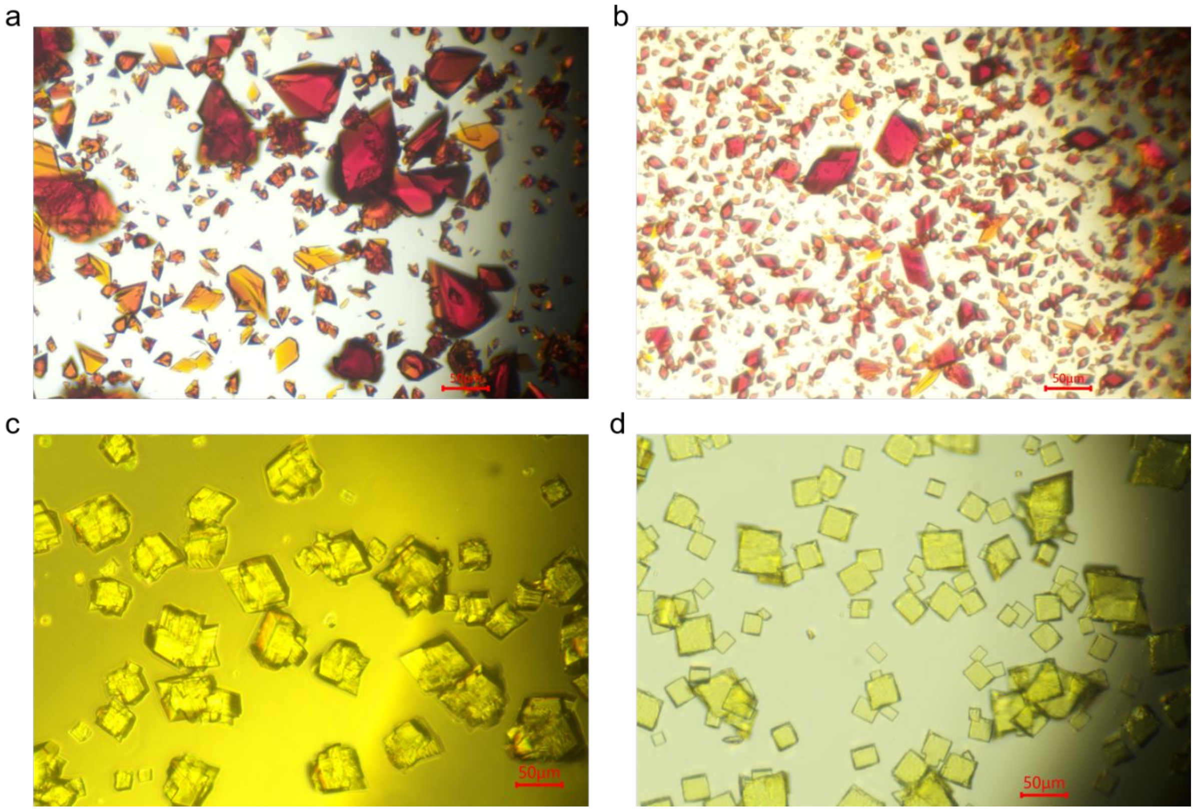

Figure S22. Microscope photos for (a) Zn_6(**M1**-(*R*, *R*))$_4$, (b) and Zn_6(**M1**-(*S*, *S*))$_4$, (c) Zn_6(**M2**-(*R*, *R*))$_4$, (d) and Zn_6(**M2**-(*S*, *S*))$_4$.

3. Crystallography Report

Table S1. SC-XRD data for Zn_6(**M1**-(*R*, *R*))$_4$ and Zn_6(**M2**-(*R*, *R*))$_4$.

Molecule	Zn_6(**M1**-(*R*, *R*))$_4$[a]	Zn_6(**M2**-(*R*, *R*))$_4$[b]
Crystallisation Solvent	DEF-CH_3CN	DEF
Space Group	*R3*	*R3*
Wavelength [Å]	Mo-Kα	Mo-Kα
Collection Temperature	100 K	100 K
Formula	$C_{168}H_{180}N_{24}O_{24}Zn_6$, 3($C_5H_{11}NO$), CH_3CN, H_2O, x(solvent)	$C_{240}H_{228}N_{24}O_{24}Zn_6$, x(solvent)
Mr	3674.08	4224.67
Crystal Size (mm)	0.304×0.089×0.031	0.030×0.020×0.020
Crystal System	trigonal	trigonal
a = *b* [Å]	20.2939(5)	39.3778(14)
c [Å]	49.1404(14)	26.9175(14)
α = *β* [°]	90	90
γ [°]	120	120
V [$Å^3$]	17526.7(10)	36147(3)
Z	3	3
D_{calcd} [g cm^{-3}]	1.044	0.582
μ [mm^{-1}]	0.666	0.326
F(000)	5784	6624
2θ range [°]	4.01–53.04	3.25–41.81
Reflections collected	150243	112527
Independent reflections, R_{int}	16038, 0.0623	16932, 0.0929
Obs. data [$I > 2\sigma$]	11766	12734
Data / restraints / parameters	16038 / 37 / 748	16932 / 820 / 886
Final R_1 values ($I > 2\sigma(I)$)	0.0543	0.0340
Final R_1 values (all data)	0.0777	0.0552
Final $wR(F^2)$ values (all data)	0.1468	0.0741
Goodness-of-fit on F^2	1.016	0.928
Largest difference peak and hole [e.A^{-3}]	0.633 / -0.263	0.115 / -0.173
CCDC	2347203	2347204

[a] Due to the severe disorder of the solvent molecules in the large crystal pores, a solvent mask implemented in Olex2[9] using the BYPASS[10] method mask was used during the final refinement cycles. The solvent mask found 184 electrons in three symmetrically equivalent 1685.5 $Å^3$ sized pores and 105 electrons in three symmetrically equivalent 287 $Å^3$ sized pores.[b] Due to the severe disorder of the solvent molecules in the large crystal pores, a solvent mask implemented in Olex2[9] using the BYPASS[10] method mask was used during the final refinement cycles. The solvent mask found 6233 electrons in an interconnected, 23718 $Å^3$ sized pore.

4. UV/Vis spectra

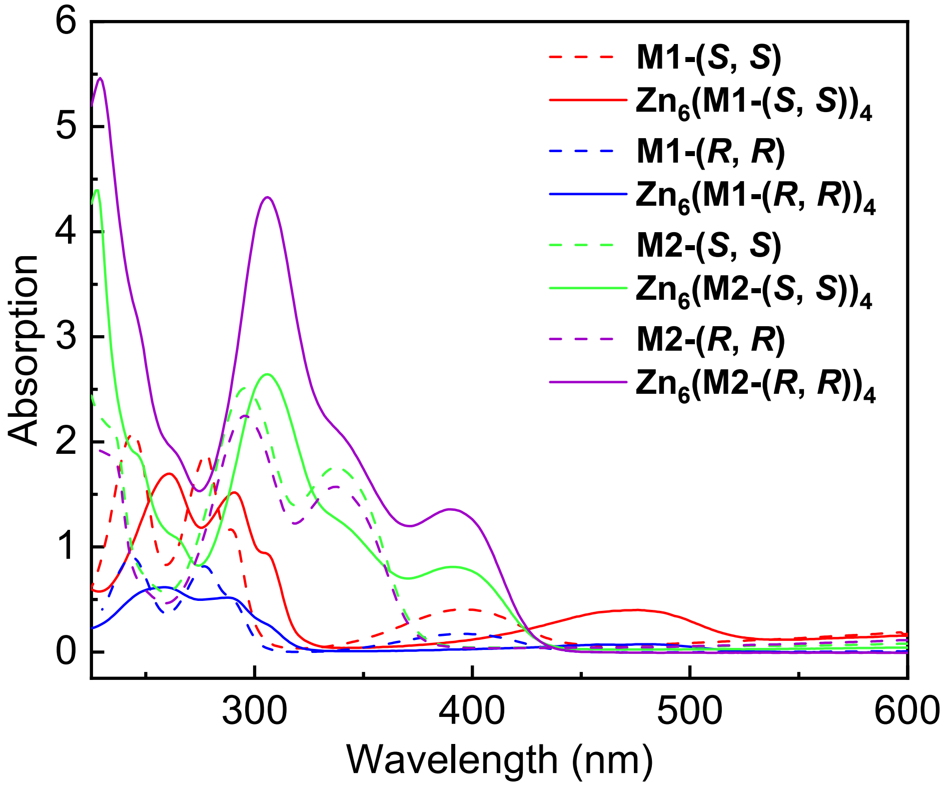

Figure S23. UV/Vis spectra of **M1, M2**, Zn_6(**M1**-(*R*, *R*))$_4$ and Zn_6(**M2**-(*R*, *R*))$_4$ in CH_2Cl_2.

5. PXRD data

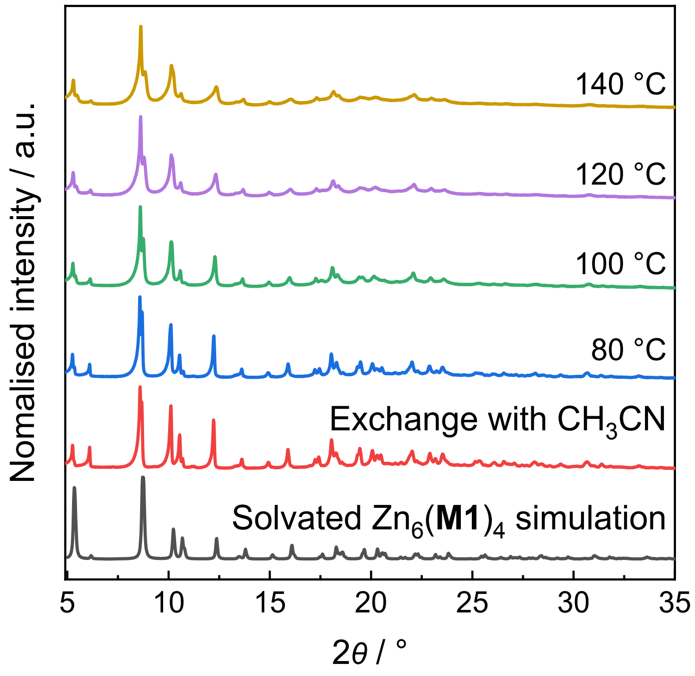

Figure S24. PXRD patterns were collected during in situ heating (298–438 K) for Zn_6(**M1**-(*R*, *R*))$_4$ exchanged with CH_3CN. The samples were loaded in borosilicate glass capillaries (diameter = 0.5 mm).

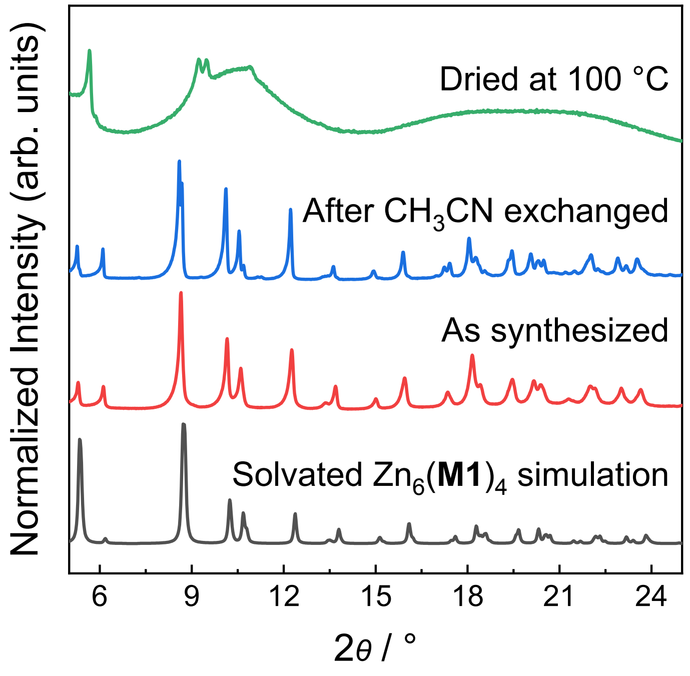

Figure S25. Simulated PXRD pattern for Zn_6(**M1**-(*R*, *R*))$_4$ using the solvated single crystal structures; PXRD patterns under different conditions: as synthesized, exchanged with CH_3CN, dried at 100 °C under a vacuum for 12 h.

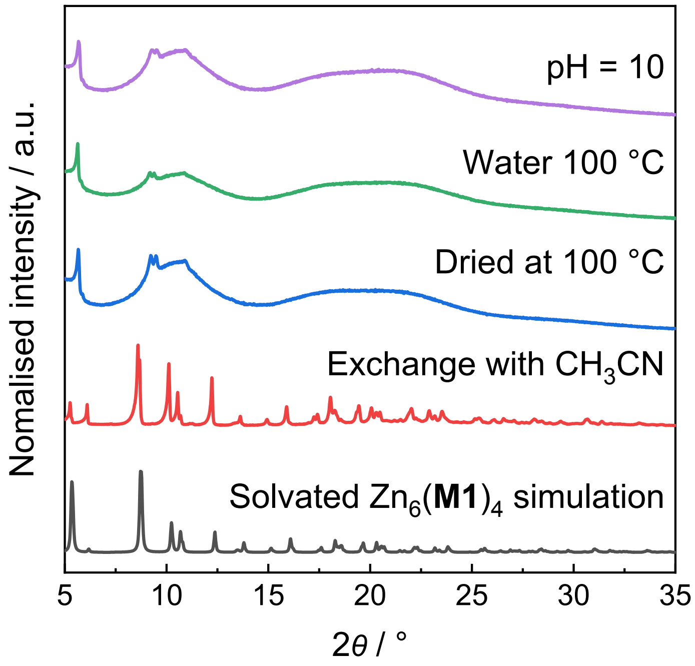

Figure S26. PXRD patterns under different conditions: exchanged with CH_3CN, dried at 100 °C under a vacuum for 12 h, boiling water (24 h), pH = 10 (24 h).

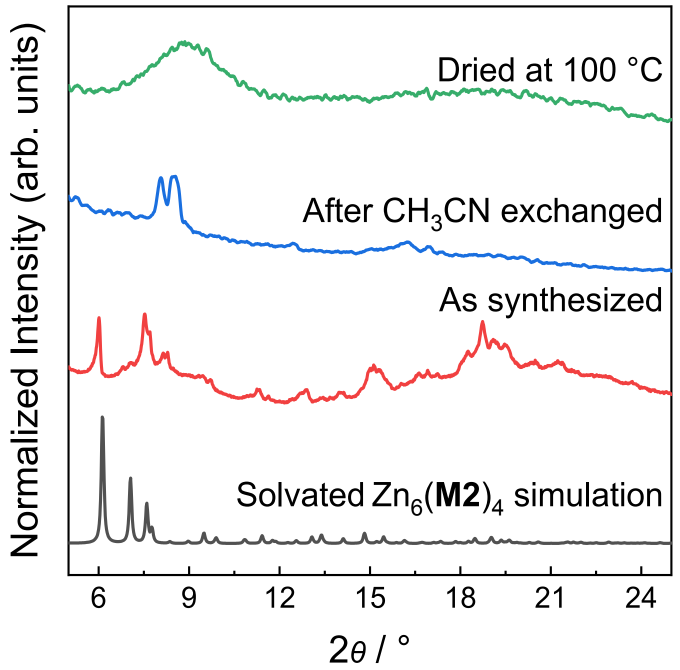

Figure S27. Simulated PXRD pattern for Zn_6(**M2**-(*R*, *R*))$_4$ using the solvated single crystal structures (bottom). PXRD patterns under three different conditions: as synthesized, exchanged with CH_3CN, and dried at 100 °C under a vacuum for 12 h.

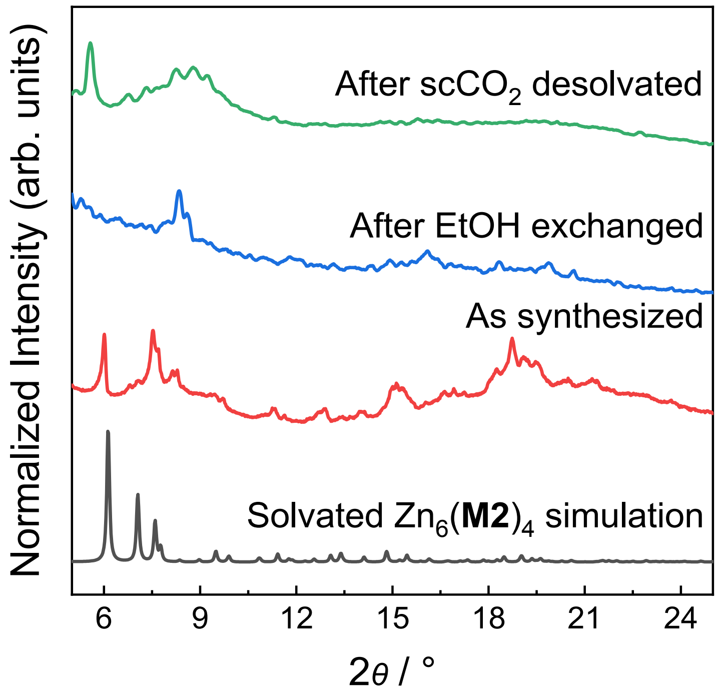

Figure S28. Simulated PXRD pattern for Zn_6(**M2**-(*R*, *R*))$_4$ using the solvated single crystal structures (bottom). PXRD patterns under three different conditions: as synthesized, exchanged with EtOH, dried by supercritical CO_2.

6. TGA data

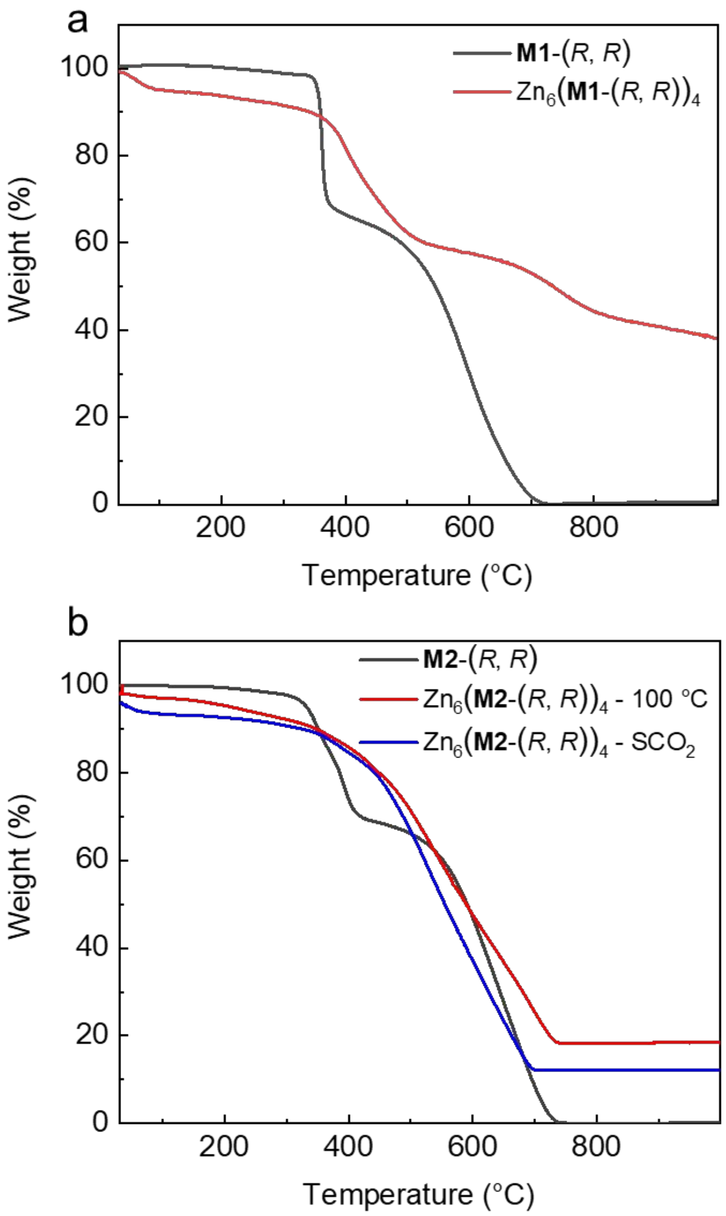

Figure S29. TGA plot of (a) **M1**-(*R*, *R*) (black line), and as-crystallized Zn_6(**M1**-(*R*, *R*))$_4$ from DEF after exchanging the DEF crystallization solvent with MeCN solvent and drying at 100 °C under vacuum for 12 h (red line). TGA plot of (b) **M2**-(*R*, *R*) (black line), as-crystallized Zn_6(**M2**-(*R*, *R*))$_4$ from DEF after exchanging the DEF crystallization solvent with MeCN solvent and drying at 100 °C in vacuum for 12 h (red line), and as-crystallized Zn_6(**M2**-(R, R))$_4$ from DEF after exchanging the DEF crystallization solvent with EtOH solvent followed by supercritical CO_2 (SCO_2) drying (blue line). All the samples were loaded onto the TGA pans in air, and we attribute the lower temperature weight losses (<100 °C) to water adsorbed by the samples before the measurements than MeCN from the solvent exchanges. The higher temperature weight losses are likely due to trace DEF solvent and water in the crystal pores. In the TGA plots, we observed far lower higher temperature weight losses for the supercritical CO_2-activated Zn_6(**M2**-(*R*, *R*))$_4$ sample than the MeCN-exchanged Zn_6(**M2**-(*R*, *R*))$_4$ sample and subsequently activated CO_2-activated Zn_6(**M2**-(*R*, *R*))$_4$ at room temperature under vacuum before performing gas sorption analysis.

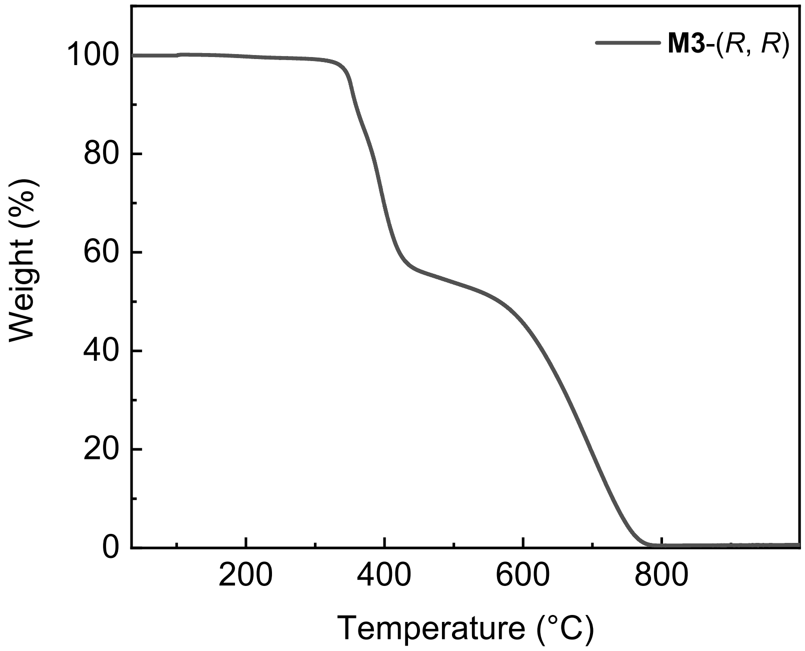

Figure S30. Thermogravimetric analysis of **M3**-(*R*, *R*) dried at 100 °C under a vacuum for 12 h prior to the TGA measurement.

7. ICP data

Table S2. ICP-OES analysis of water and 0.1 M HCl (aq) solutions that had been used to immerse the solid samples of Zn_6(**M1**-(*R*, *R*))$_4$ and Zn_6(**M2**-(*R*, *R*))$_4$ for 10 days at room temperature. We used the ICP values as markers to determine if the solid samples of Zn_6(**M1**-(*R*, *R*))$_4$ and Zn_6(**M2**-(*R*, *R*))$_4$ decomposed during these experiments. The analysis was conducted after filtering the solutions to remove the remaining solids, and the resulting filtrates were diluted with water before the measurement. Each measurement was performed at three different wavelengths (202.548 nm, 213.857 nm, and 472.215 nm). Each wavelength was calibrated beforehand using four standards, and the relative standard deviations (% RSDs) were determined from these calibration measurements.

Name	Element Label (nm)	Concentration (ppm)	%RSD
Zn_6(**M1**-(*R*, *R*))$_4$ (water)	Zn (202.548 nm)	2.32	2.87
Zn_6(**M1**-(*R*, *R*))$_4$(water)	Zn (213.857 nm)	2.24	1.13
Zn_6(**M1**-(*R*, *R*))$_4$(water)	Zn (472.215 nm)	2.30	5.02
Zn_6(**M2**-(*R*, *R*))$_4$(water)	Zn (202.548 nm)	4.36	1.52
Zn_6(**M2**-(*R*, *R*))$_4$(water)	Zn (213.857 nm)	4.30	1.42
Zn_6(**M2**-(*R*, *R*))$_4$(water)	Zn (472.215 nm)	4.38	3.58
Zn_6(**M1**-(*R*, *R*))$_4$ (pH = 1)	Zn (202.548 nm)	65.33	5.06
Zn_6(**M1**-(*R*, *R*))$_4$(pH = 1)	Zn (213.857 nm)	59.93	0.66
Zn_6(**M1**-(*R*, *R*))$_4$(pH = 1)	Zn (472.215 nm)	62.35	1.97
Zn_6(**M2**-(*R*, *R*))$_4$(pH = 1)	Zn (202.548 nm)	56.98	1.04
Zn_6(**M2**-(*R*, *R*))$_4$(pH = 1)	Zn (213.857 nm)	55.12	0.94
Zn_6(**M2**-(*R*, *R*))$_4$(pH = 1)	Zn (472.215 nm)	57.49	1.53
Zn_6(**M1**-(*R*, *R*))$_4$ (calculated)	--	59.30	--
Zn_6(**M2**-(*R*, *R*))$_4$ (calculated)	--	46.40	--

8. Gas sorption

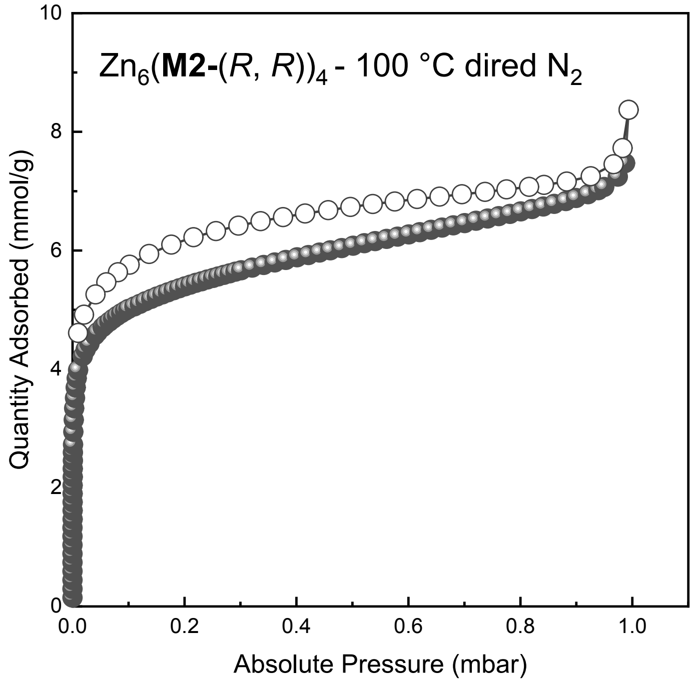

Figure S31. N_2 sorption isotherms (77 K) of Zn_6(**M2**-(*R*, *R*))$_4$ exchanged with CH_3CN, dried at 100 °C in vacuum for 12 h.

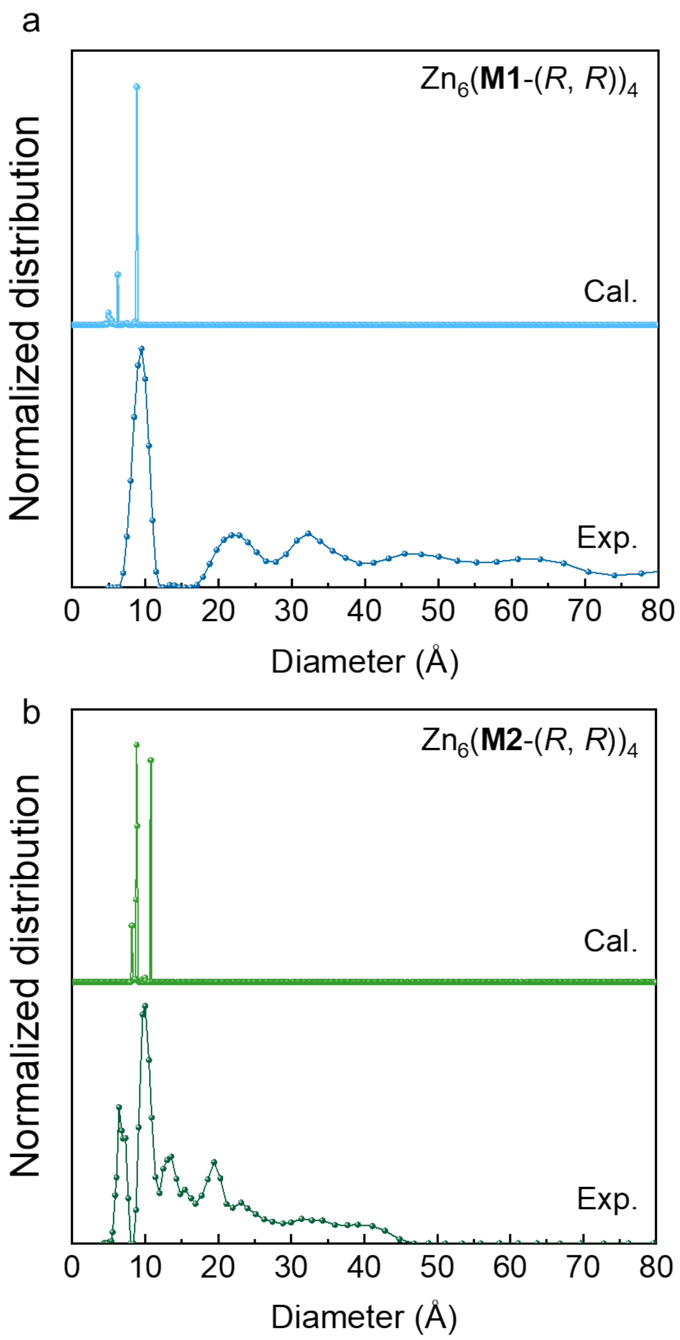

Figure S32. (a) Pore size distribution histograms for Zn_6(**M1**-(*R*, *R*))$_4$. (b) Pore size distribution histograms for Zn_6(**M2**-(*R*, *R*))$_4$ activated by supercritical CO_2.

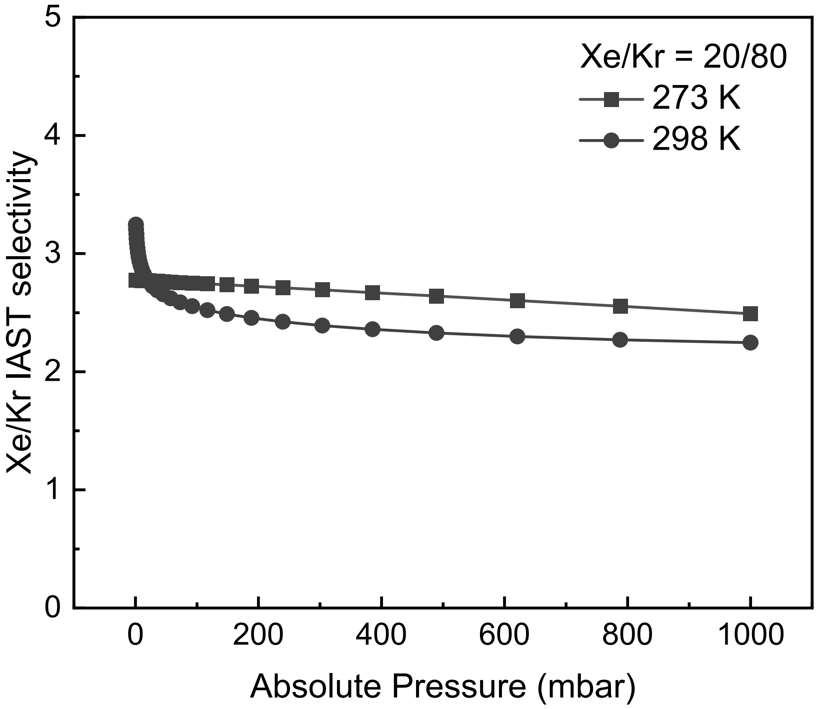

Figure S33. IAST selectivity of Xe/Kr = 20/80 mixtures for Zn_6(**M1**-(*R*, *R*))$_4$ as calculated from the pure gas sorption isotherms.

9.0 References

(1) Caldeweyher, E.; Ehlert, S.; Hansen, A.; Neugebauer, H.; Spicher, S.; Bannwarth, C.; Grimme, S., A Generally Applicable Atomic-Charge Dependent London Dispersion Correction. *J. Chem. Phys.* **2019,** *150*, 154122.

(2) Bannwarth, C.; Caldeweyher, E.; Ehlert, S.; Hansen, A.; Pracht, P.; Seibert, J.; Spicher, S.; Grimme, S., Extended Tight-Binding Quantum Chemistry Methods. *WIREs Comput. Mol. Sci.* **2021,** *11*, e1493.

(3) Grimme, S.; Bannwarth, C.; Shushkov, P., A Robust and Accurate Tight-Binding Quantum Chemical Method for Structures, Vibrational Frequencies, and Noncovalent Interactions of Large Molecular Systems Parametrized for All Spd-Block Elements (Z= 1–86). *J. Chem. Theory Comput.* **2017,** *13*, 1989-2009.

(4) Bannwarth, C.; Ehlert, S.; Grimme, S.; Tight-Binding, B. P. S.-C., Quantum Chemical Method with Multipole Electrostatics and Density-Dependent Dispersion Contributions. *J. Chem. Theory Comput.* **2019,** *15*, 1652-71.

(5) Ryckaert, J.-P.; Ciccotti, G.; Berendsen, H. J. C., Numerical Integration of the Cartesian Equations of Motion of a System with Constraints: Molecular Dynamics of N-Alkanes. *J. Comput. Phys.* **1977,** *23*, 327-341.

(6) Berendsen, H. J. C.; Postma, J. P. M.; Gunsteren, W. F. v.; DiNola, A.; Haak, J. R., Molecular Dynamics with Coupling to an External Bath. *J. Chem. Phys.* **1984,** *81*, 3684-3690.

(7) Miklitz, M.; Jelfs, K. E., Pywindow: Automated Structural Analysis of Molecular Pores. *J. Chem. Inf. Model.* **2018,** *58*, 2387-2391.

(8) Szymkowiak, J.; Kwit, M., Electronic and Vibrational Exciton Coupling in Oxidized Trianglimines. *Chirality* **2018,** *30*, 117-130.

(9) Dolomanov, O. V.; Bourhis, L. J.; Gildea, R. J.; Howard J. A. K.; Puschmann, H., OLEX2: A Complete Structure Solution, Refinement and Analysis Program. *J. Appl. Cryst.* **2009**, *42*, 339-341.

(10) P. van der Sluis, P.; Spek A. L., BYPASS: An Effective Method for the Refinement of Crystal Structures Containing Disordered Solvent Regions. *Acta Cryst.* **1990**, *A46*, 194-201.
